# Accurate MRF‐Based 3D Multi‐Channel B_1_^+^ Mapping in the Human Body at 7 T

**DOI:** 10.1002/nbm.70080

**Published:** 2025-06-18

**Authors:** Max Lutz, Sebastian Flassbeck, Christoph Stefan Aigner, Felix Krueger, Tobias Schaeffter, Sebastian Schmitter

**Affiliations:** ^1^ Physikalisch‐Technische Bundesanstalt Braunschweig and Berlin Germany; ^2^ Bernard and Irene Schwartz Center for Biomedical Imaging, Department of Radiology New York University Grossman School of Medicine New York New York USA; ^3^ Center for Advanced Imaging Innovation and Research (CAI^2^R), Department of Radiology New York University Grossman School of Medicine New York New York USA; ^4^ Einstein Center Digital Future Berlin Germany; ^5^ Department of Biomedical Engineering Technical University of Berlin Berlin Germany; ^6^ Center for Magnetic Resonance Research University of Minnesota Minneapolis Minnesota USA; ^7^ Medical Physics in Radiology German Cancer Research Center (DKFZ) Heidelberg Germany

**Keywords:** 7 Tesla, B1+ mapping, body MRI, MRF, ultrahigh field MRI

## Abstract

This work proposes a 3D multi‐transmit channel B_1_^+^ mapping approach based on magnetic resonance fingerprinting (MRF) for the human abdomen at 7 T. A stack‐of‐stars acquisition is employed to achieve motion‐robust 3D encoding, along with a hybrid method where transmit (Tx) channel‐wise B_1_^+^ information is obtained through low flip angle GRE images. B_1_^+^ mapping at ultra‐high field (UHF) in the human abdomen is particularly challenging due to the large dynamic range of B_1_^+^, the extensive field of view (FOV), and the effects of respiratory motion. Few methods have been proposed to address these challenges, with a significant limitation being the relatively low RF power available at UHF, especially for pTx systems with a 8 × 1 kW power configuration. This limitation makes it difficult to achieve FAs greater than 30° in central body regions, which are required for accurate results with classical methods. In contrast, Tx channel‐combined MRF‐based B_1_^+^ mapping has been validated as accurate for FAs greater than 6°, offering improved accuracy at low FAs. Here, two Tx channel‐combined MRF‐based B_1_^+^ maps (B1‐MRF) are acquired using two tailored complementary phase shims to obtain absolute B_1_^+^ information across the entire FOV. The 3D hybrid approach was validated against a 2D reference using phantoms and in vivo free‐breathing scans in three subjects with varying BMIs, where only one Tx channel was active at a time. The comparison showed strong agreement, with the 3D hybrid acquisition demonstrating improved performance in regions affected by flow, low FAs, or low signal‐to‐noise ratio compared to the 2D implementation. The higher accuracy and level of detail provided by the proposed method, in contrast to existing methods, are particularly relevant for several applications, including the validation of faster approaches, validation of electromagnetic simulations (which are safety‐critical), and the creation of B_1_^+^ map libraries for applications such as AI‐based B_1_^+^ mapping or universal pulse calculations.

## Graphical abstract

A 3D hybrid multi‐channel B_1_^+^ mapping approach based on MR Fingerprinting was implemented and validated at 7 T, demonstrating high accuracy and detail in the resulting B_1_^+^ maps. Compared to existing approaches, the proposed method offers an increased dynamic FA range and higher accuracy in low FA regions.

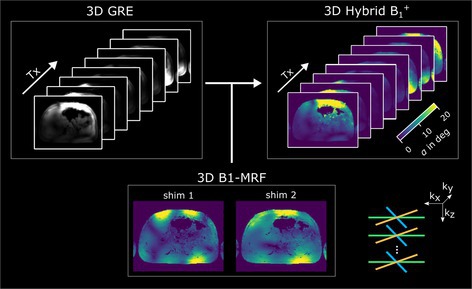

**Abbreviations**
B_1_^+^
: transmit magnetic field
BMI: body mass index
EM: electromagnetic
FA: flip angle
FFT: fast Fourier transform
FOV: field of view
gEPT: gradient‐based electrical property tomography
MRF: magnetic resonance fingerprinting
pTx: parallel transmission
PVP: polyvinylpyrrolidone
ROI: region of interest
SAR: specific absorption rate
SOM: sum of magnitudes
TA: acquisition time
TBWP: time bandwidth product
TF: time frame
Tx: transmit
UHF: ultra‐high field

## 1 Introduction

Ultra‐high field (UHF) MRI at 7 T and above offers several advantages compared to lower fields, particularly an increased SNR [1]. However, UHF MRI also faces substantial challenges due to inhomogeneous transmit (Tx) magnetic fields (B_1_^+^) caused by the increased Larmor frequency. This non‐uniformity leads to spatially varying flip angles (FA), causing spatially varying contrast and “drop‐out areas” in large fields of views (FOVs) like the human abdomen [2, 3, 4], potentially limiting clinical feasibility. While the spatial B_1_^+^ inhomogenieties can be addressed by parallel transmission (pTx) [5, 6], precise knowledge of the Tx channel‐wise B_1_^+^ distribution is essential not only for pTx but also for applications such as electrical property tomography (EPT), specifically gradient‐based EPT (gEPT) [7, 8]. Measuring channel‐wise B_1_^+^ distributions in the human abdomen at UHF is particularly challenging due to the large dynamic range of B_1_^+^ across the body, as well as additional factors such as respiratory motion.

Few approaches for channel‐wise absolute B_1_^+^ mapping in the human body at UHF were proposed [9, 10, 11] and are listed in Table 1. While these methods enable the acquisition of absolute channel‐wise B_1_^+^ maps, their accuracy may be limited on systems with constrained RF power or less efficient Tx arrays. Even with all channels being active and two complementary shims, large organs like the liver often contain regions where achieving a sufficiently high FA is challenging. In such areas, the accuracy of the underlying absolute B_1_^+^ mapping methods (DREAM [12], satTFL [13], sandwich satTFL [14], AFI [15]) becomes increasingly biased, as FAs greater than approximately 30° would be required for high accuracy [16, 17, 18]. While a more detailed analysis of the accuracy would require in vivo validations with a reference method, such an analysis is challenging, since currently no channel‐wise 3D B_1_^+^ mapping method for UHF body imaging exists that provides accurate maps with high dynamic range and high spatial resolution within a few minutes of acquisition time. For example, Kent et al. [11] noted in their discussion “the lack of a reliable ground truth in the body” [11]. Due to this limitation, all of the approaches mentioned above have not been experimentally validated in vivo (cf. Table 1). Additionally, all approaches in Table 1 have been acquired during breath‐hold, which limited so far the ability to achieve B_1_^+^ maps with in‐plane resolutions of less than 6 mm. Although coarser maps are generally sufficient for pTx calibration, applications such as in vivo validations of EM simulations [19, 20, 21, 22] or gEPT [7, 8] typically require higher‐resolution B_1_^+^ maps. Furthermore, with the transition to higher field strengths such as 11.7 T or 14 T, higher spatial B_1_^+^ resolution may become necessary for accurate pTx calibration due to the further shortening of the RF wavelength. Although a more accurate method with in‐plane resolution below 6 mm leads to longer acquisition times that limit routine clinical use, it has still high value as a reference standard for evaluating faster approaches. Moreover, it offers significant advantages in scenarios where mapping accuracy across the entire FOV or large organs is prioritized over acquisition time, such as in building libraries for universal pulse design [23, 24] or generating training datasets for AI‐based B_1_^+^ calibration [25].

**TABLE 1:** Overview of existing approaches to map the channel‐wise B_1_^+^ distribution in the human body at 7 T.

	Fourier phase‐encoded DREAM [9]	B1TIAMO [10]	Sandwich TxLR [11]
Tx encoding	Interferometric	Hybrid	Hybrid
Absolute B_1_^+^ mapping	DREAM [12]	satTFL [13]	Sandwich satTFL [14]
Dimensionality	2D mutlislice	2D	3D
Resolution	6 mm iso	6 × 6 × 20 mm^3^	15.8 × 15.8 × 11.9 mm^3^
Targeted organ body 7 T	Liver	Kidneys	Heart
Acquisition time	13 × 12.6 s (13 breath‐holds)	16 s	23 heartbeats
Mapped Tx channels	8	32	8
Validation	None	Phantom Simulations	Simulations

We recently investigated a 2D MRF‐based B_1_^+^ mapping approach (B1‐MRF) [18] that is accurate and precise, even at low FAs, with a dynamic range of 6°–74° and B_1_^+^ errors below 5%. This enhanced dynamic range, in principle, allows for direct B_1_^+^ mapping of the individual Tx channels, providing accurate, high‐resolution B_1_^+^ maps of each Tx channel. Although scan time is not limited by breath‐hold requirements due to the non‐Cartesian trajectory of this method, mapping 8Tx channels requires 5 min per slice, and extending the method to multiple slices to cover a 3D volume would result in scan times beyond 1 h. Therefore, a faster approach that preserves B_1_^+^ accuracy is needed, which can be achieved through a hybrid method.

In this work, we develop and validate a 3D Tx channel‐wise hybrid B_1_^+^ mapping approach based on B1‐MRF, offering high accuracy and precision across the expected large dynamic FA range in the human body at 7 T. The 3D acquisition is achieved through stack‐of‐stars encoding [26], enabling free‐breathing scans with a resolution of 2 × 2 × 6 mm^3^. Channel‐wise B_1_^+^ mapping is achieved by incorporating a low FA GRE acquisition for each Tx channel into the stack‐of‐stars sequence, with a hybrid combination following the B1TIAMO [10] approach. Two complementary shims were pre‐calculated using an adapted AMORE [27] cost function. This 3D hybrid method is compared to single‐channel 2D B1‐MRF [18] mapping in phantom and in vivo scans using an 8Tx body coil.

## 2 Methods

### 2.1 Workflow

**FIGURE 1:**
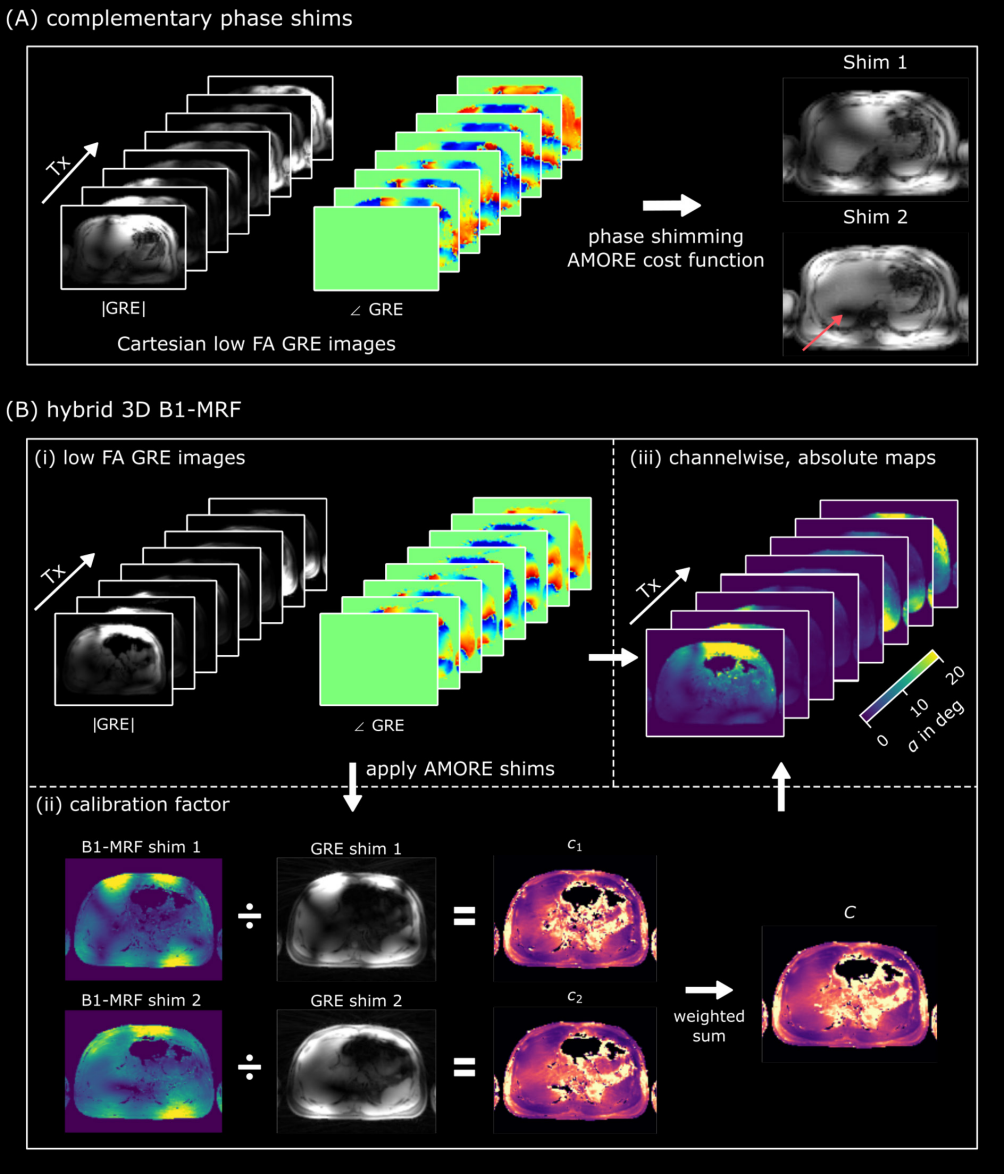
Workflow of the proposed 3D hybrid approach. (A) Illustration of the AMORE shim calculation, where 2D low FA GRE images from six slices are used to calculate two complementary shims. The red arrow indicates a drop‐out region in one shim for the liver, where using only this shim would lead to biased results in this region. A B1‐MRF‐based B_1_^+^ map with the second complementary shim is acquired to address this issue as proposed by B1TIAMO. These shims are then loaded into the hybrid 3D B1‐MRF sequence (B). (i) The data from the first part of the proposed sequence, where low FA GRE images are acquired for each channel. In (ii), the absolute B_1_^+^ map is obtained from the second part of the proposed sequence with the two AMORE shims. After image reconstruction, the combination to obtain the channel‐wise maps is performed in a B1TIAMO fashion, where the same shims are applied to the GRE images, and calibration factors c_1_ and c_2_ are calculated. Finally, the shim‐wise calibration factors c_1_ and c_2_ are merged by weighting them quadratically with the FA value from the absolute maps to a single calibration factor C. Multiplying the calibration factor C with the GRE images from (i) results in channel‐wise absolute B_1_^+^ maps (iii).

The proposed workflow is illustrated in Figure 1. The technique employs a hybrid approach, where channel‐wise low FA GRE images are linearly combined with two absolute, channel‐combined MRF‐based B_1_^+^ maps, as proposed by B1TIAMO [10]. Note that a single absolute map is insufficient, as each map typically contains regions with B_1_^+^ dropouts (cf. Figure 1A, red arrow). These dropout regions are then addressed by the second B_1_^+^ map acquired with an orthogonal shim. In principle, two B_1_^+^ shim sets, such as CP^+^ or CP^2+^, could be used. However, due to the non‐circular shape of the body and arrangement of the coil elements, the orthogonality of the two modes is not guaranteed. As a result, two complementary, tailored phase shims were calculated for each subject using the AMORE cost function [27].

### 2.2 RF Phase Shimming

The complementary shim sets were generated from a quick calibration scan (cf. Figure 1A) using 2D multislice GRE images in a low FA regime. These GRE images were acquired eight times during a single breath‐hold, with only one Tx channel active [28]. The acquisition parameters are provided in Table 2. The acquired data was then exported for phase shimming. Phase shimming was performed using the AMORE cost function [27], which was slightly modified to penalize only low values across both shims, while values above a certain threshold were not penalized.

**TABLE 2:** Acquisition parameters of phantom and in vivo studies with TR, TE, acquisition time (TA), flip angle (FA), slice thickness, FOV and pixel/voxel size, MRF cycles.

	Cartesian low FA GRE images	3D hybrid B1‐MRF	2D 1Tx B1‐MRF
TR/ms	5.39	4.75	4.4
TE/ms	2.84	2.1	2.2
TA	37 s	9 min 36 s	4 min 56 s (per orientation)
Number of included measurements	10 (all Tx, noise, Tx 1–8)	GRE: 8 (Tx 1–8) MRF: 2 (AMORE shims)	8 (Tx 1–8)
Nominal FA $\alpha_{\mathrm{nom}}$/°	20	GRE: 15 MRF: 55	60
Slice thickness/mm	6	—	5
Number of slices	6	30	1
Slice orientation	Transversal	Transversal	Transversal/coronal/sagittal
FOV/mm^2^ or mm^3^	384 × 254	384 × 384 × 180	384 × 384
Pixel/voxel size/mm^2^ or mm^3^	4 × 4	2 × 2 × 6	2 × 2
MRF cycles	—	60 (per MRF shim)	13 (for each measurement)

Since phase shim optimization is a non‐convex problem, 100 random initial shim pairs were used, and the solution with the lowest function value after optimization was selected. For the optimization process, the *optimize.minimize* function from the *SciPy* [29] library was used with the *L‐BFGS‐B* [30, 31] solver applied and bounds of [0, 2π] set for the phase of the shims. Shimming took 17 s on an *Intel Xeon E5‐2687W v4* system with 2x24 cores and 1 TB RAM.

The resulting AMORE shims were then written to a text file, exported to the scanner and read in by the hybrid 3D B1‐MRF sequence.

### 2.3 Sequence Implementation

#### 2.3.1 General

The MRF‐based sequence was implemented with slab‐selective excitation and 3D stack‐of‐stars encoding. The sequence builds upon previous work [18] and is FLASH‐based, with no magnetization preparation, such as inversion pulses, applied to reduce T_1_ sensitivity. Sinc‐shaped RF pulses with a duration of 1.33 ms and a time‐bandwidth product (TBWP) of 4 were utilized. An RF quadratic spoil increment of 84° was applied, and gradient spoiling was implemented to achieve 8π dephasing across one encoded slice. Further acquisition parameters are listed in Table 2. The sequence consists of two main parts, with a total acquisition time (TA) of 9 min 36 s, acquired in the following order: (i) First, channel‐wise low FA GRE images are acquired (TA = 3 min 48 s), followed by (ii) the acquisition of two absolute, MRF‐based, channel‐combined B_1_^+^ maps using the two AMORE phase shims to provide absolute B_1_^+^ information (TA = 5 min 48 s).

#### 2.3.2 3D Encoding

**FIGURE 2:**
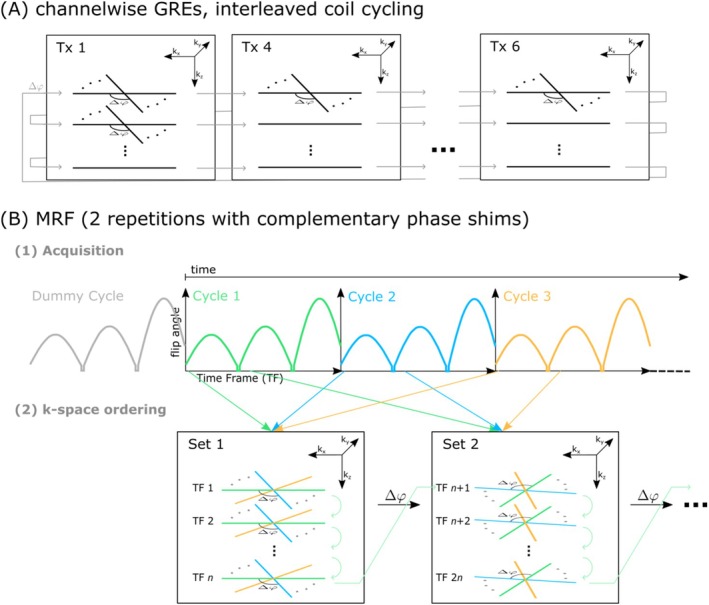
Overview of the 3D hybrid stack‐of‐stars B1‐MRF acquisition and the corresponding k‐space ordering. (A) The k‐space ordering of the 3D channel‐wise GRE images where a constant FA is used. The interleaved coil cycling technique is applied, where the same spoke is successively acquired for all Tx channels in the order: Tx 1, Tx 4, Tx 3, Tx 7, Tx 8, Tx 5, Tx 2, Tx 6. Once the last Tx channel is reached, the partition index is increased, and the readout angle remains constant. Upon reaching the last partition, the first partition is sampled again, and the readout angle is incremented by a golden angle of 111.25°. (B) The ordering of the MRF part of the sequence. (1) The continuously played‐out FA pattern across 60 Cycles, with the sequence based on FLASH and no preparation pulses (e.g., inversion pulses) applied. A dummy cycle precedes cycle 1 to ensure identical magnetization at the start of each subsequent cycle. Each cycle consists of 600 time frames (TF). In (2) the k‐space order is illustrated. A total of 20 sets are acquired, each containing *n* = 30 TFs, corresponding to the number of partitions. The partition index is incremented first from one TF to the next within the same cycle. When the last partition is reached, the data is binned into the second set, and the readout angle is incremented. For the second cycle, the first 30 TFs are again binned into set 1, but the readout angle is increased by the golden angle. From the last cycle of one set to the next, the readout angle is similarly incremented by a golden angle. The readout angle is thus defined as: $\theta =\left(i_{\mathrm{CYC}}+i_{\mathrm{set}}\cdot N_{\mathrm{CYC}}\right)\cdot 111.25^{{}^{\circ}}$, where $i_{\mathrm{CYC}}$ and $i_{\mathrm{set}}$ denote the indices of the cycle and Set, respectively, and $N_{\mathrm{CYC}}$ is the total number of cycles.

Gradients along the z‐dimension were applied to extend the sequence to 3D encoding. In the following, *slab* refers to the coverage of the object along the z‐dimension in image space, subdivided into *slices*, while *partition* refers to the individual phase‐encoding steps of the stack‐of‐stars acquisition along the k_z_‐dimension in k‐space. It has been demonstrated that acquiring different partitions in quick succession before changing the readout angle improves motion robustness in a stack‐of‐stars acquisition, compared to sampling one partition entirely before increasing the partition index [26]. Consequently, the partition index is changed before the readout angle in this implementation, as illustrated in Figure 2. Sampling the partition direction first during the transient MRF signal (cf. Figure 2B) results in contrast mixing along the partition dimension, as a single set used for reconstruction contains different time frames with varying image intensities. However, previous work has demonstrated that this contrast mixing has a minor influence when the FAs change only smoothly [32].

#### 2.3.3 GRE Part

For the channel‐wise GRE acquisition, the interleaved coil cycling technique [10] was used, where the same spoke is acquired for each Tx channel before changing the partition index as illustrated in Figure 2A. The order of Tx channels follows Tx 1, Tx 4, Tx 3, Tx 7, Tx 8, Tx 5, Tx 2, and Tx 6 to minimize the consecutive acquisition of adjacent coil elements. Once the last partition is reached, the readout angle is incremented by a golden angle of 111.25°. Two hundred radial spokes were acquired per partition and Tx channel.

#### 2.3.4 MRF Part

The acquisition and k‐space ordering for the B1‐MRF part are illustrated in Figure 2B. Similar to the 2D implementation [18], 600 time frames (TFs) were used, with each TF corresponding to one TR, and the FA pattern from Jiang et al. [33] was applied. The FA pattern was repeated 60 times per shim setting, where each repetition is referred to as a cycle in the following. Before the first cycle, a dummy cycle was played out to ensure identical magnetization at the start of each subsequent cycle [18, 34]. Thirty partitions were used, where the partition index was varied within one cycle. The data was binned into sets, as suggested by Riel et al. [32], with each set consisting of 30 TFs. Once the last partition was reached, the data was binned into the next set, and the readout angle was incremented by a golden angle. The readout angle is defined as: $\theta =\left(i_{\mathrm{CYC}}+i_{\mathrm{set}}\cdot N_{\mathrm{CYC}}\right)\cdot 111.25^{{}^{\circ}}$, where $i_{\mathrm{CYC}}$ and $i_{\mathrm{set}}$ denote the indices of the cycle and set, respectively, and $N_{\mathrm{CYC}}$ is the total number of cycles.

### 2.4 Dictionary Calculation

Dictionary calculation was based on Bloch simulations, where 33,824 unique fingerprints were generated. Discrete T_1_ values ranged from 50 to 3446 ms, with each T_1_ value incremented by 8% from the previous one. B_1_^+^ was implemented as a relative weighting factor of the nominal FAs (0°–55°), ranging from 0.01 to 1.5 in steps of 0.01.

**FIGURE 3:**
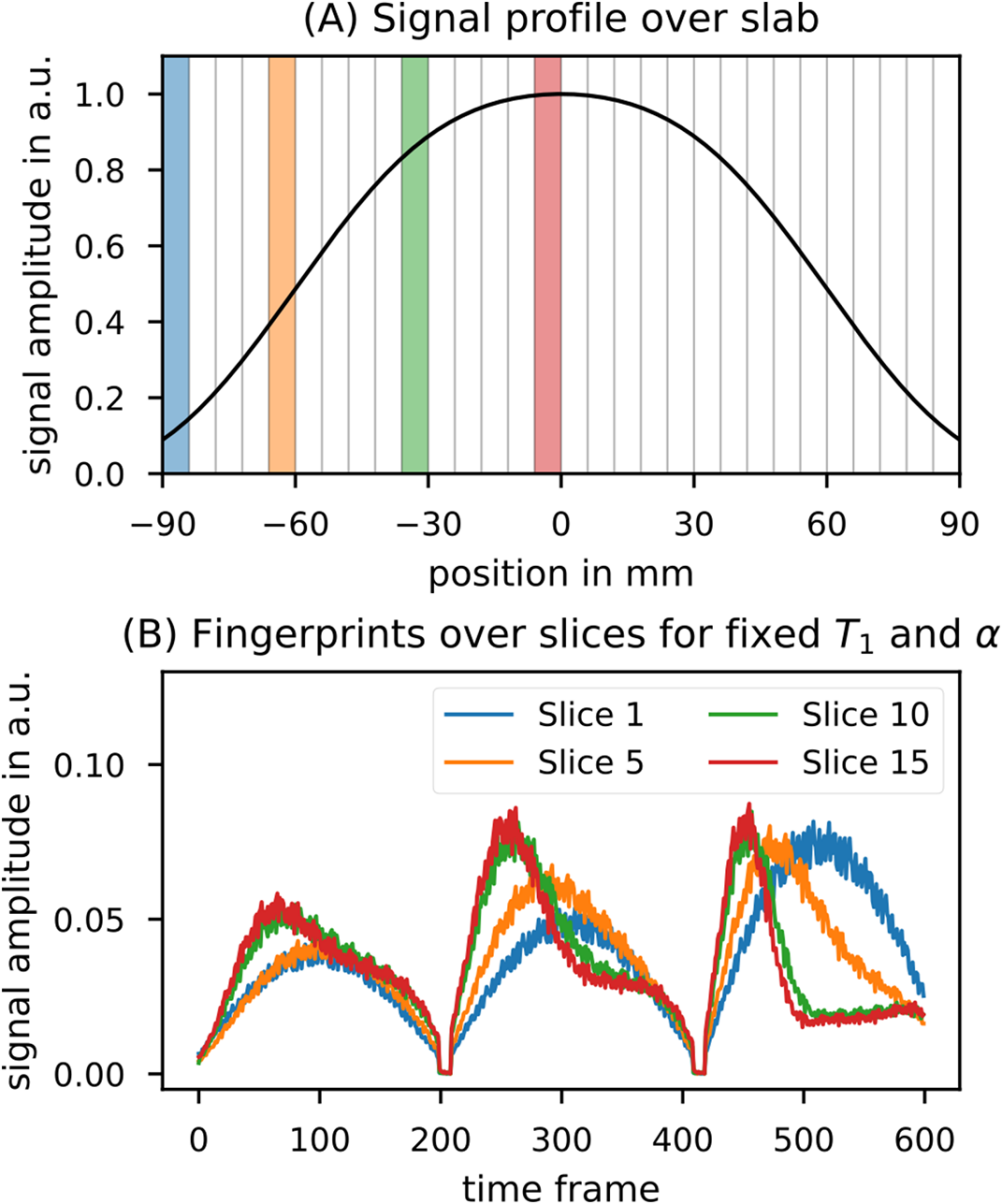
Compartmentalization of the MRF dictionary depending on the position of the slice within the slab. (A) The slab profile, where the signal intensity varies depending on the slice position. The range is matched to the experimental setup, with the nominal slab thickness spanning from −60 to 60 mm (20 slices), with an additional 30 mm oversampled on each side (10 slices). (B) The resulting fingerprints for the different slices for a fixed T_1_ = 1.3 s and α = 30°. Here, α refers to the maximum FA during the MRF pattern for the central slice, and the dictionary inherently corrects α for slices that are not in the center of the slab profile. The fingerprints show a strong dependence on the position within the slab, as the slab profile deviates substantially from a rectangular shape.

Due to varying contrast induced by the slab profile along the slab dimension for constant T_1_/B_1_^+^ values, the Bloch simulation signal was split into slices along the excited slab, and a separate dictionary was created for each slice, as illustrated in Figure 3. Figure 3 also demonstrates how the signal curve varies for fixed T_1_ and B_1_^+^ values due to the slab profile depending on the position within the slab. During the MRF matching step, the slice position was considered by using the appropriate dictionary for each respective slice.

The simulation included 4001 isochromats along the slab dimension, and the resulting signal was then divided into the corresponding slices. The simulations were conducted with 50% oversampling, meaning an additional 25% was sampled beyond the nominal slab thickness on both sides, aligning with the measurements. Since the FA pattern is played out continuously, two cycles were simulated. The signal curve from the first cycle was not stored in the dictionary and was only used to obtain the initial magnetization for the start of the subsequent cycles, which was assumed to be identical for all consecutive cycles. The dictionary was then created based on the simulation of the FA pattern using this initial magnetization. The 600 TFs from the simulation were grouped into 20 different sets, with 30 TFs per set to match the measurements (cf. Figure 2). The 30 TFs within each set were averaged to obtain the dictionary signals, and all dictionary entries were normalized to have a sum of squared magnitude of 1.

### 2.5 Image Reconstruction

Reconstruction was performed offline in *Python* on an *Intel Xeon E5‐2687W v4* system with 2 × 24 cores and 1 TB RAM, with separate reconstructions for the two parts of the acquisition: the 3D GRE images and the two 3D MRF B_1_^+^ maps. For both parts, a fast Fourier transform (FFT) was first applied along the partition dimension, and the individual slices were then reconstructed using a nonuniform FFT based on FINUFFT [35, 36]. For the 3D low FA GRE image reconstruction, an iterative SENSE reconstruction [37] with eight conjugate gradient iterations was implemented. In contrast, no iterative scheme was applied to the image reconstruction of the two absolute B_1_^+^ maps. Data from the same set of different cycles were binned into a single k‐space (cf. Figure 2). Receive coil sensitivity maps for coil combination were obtained using a singular value decomposition (SVD)‐based approach [38].

MRF matching was performed by calculating the dot product between the measured signal and all dictionary entries [39] for the respective slice. The effect of the slab profile (e.g., as shown in Figure 3, where the actual FA at off‐center positions is lower) is inherently corrected by the matching scheme, as it returns only a relative value. The ratio remains unaffected when both actual and nominal FAs are lower. Each voxel was then assigned the B_1_^+^ and T_1_ values corresponding to the dictionary entry with the highest dot product match.

### 2.6 Hybrid Combination

After reconstruction, the channel‐wise 3D GRE images and the channel‐combined 3D B_1_^+^ maps were merged as proposed by B1TIAMO [10] to obtain channel‐wise absolute B_1_^+^ maps, as illustrated in Figure 1B. A calibration factor for each voxel for both shims was calculated by dividing the absolute B_1_^+^ value from the MRF‐based acquisition for each shim by the corresponding channel‐wise GRE images with applied phase shim. The two resulting calibration factor maps were then combined by weighting them quadratically with the absolute B_1_^+^ value for the respective shim, as described in B1TIAMO [10].

### 2.7 Imaging Experiments

Phantom and in vivo scans were performed on a 7 T scanner (Magnetom 7 T, Siemens Healthineers, Erlangen, Germany) equipped with an eight‐channel pTx system and 8 x 1 kW RF amplifiers (Stolberg AG, Stolberg, Germany). The scans were performed with an 8Tx/16Rx body coil [21] consisting of 8 dipole and 8 loop elements, where only the 8 dipoles were used for transmission, and all 16 elements were used for reception. For the phantom scans, a body phantom consisting of polyvinylpyrrolidone (PVP) with a PVP/H_2_O ratio of 1.03 was used, with NaCl added at a ratio of 0.051 (NaCl/H_2_O) yielding measured values of ε_r_ = 44 and σ = 0.64S/m at 298 MHz. In vivo scans targeting the abdomen were performed according to an approved Institutional Review Board protocol, and three healthy subjects were scanned after informed consent (2 male, 1 female, 28–35 years, BMI = 18.1–27.8 kg/m^2^) under free‐breathing.

As a reference for the 3D hybrid scans, the 2D B1‐MRF sequence was used [18], as validation measurements against precise reference methods demonstrated high accuracy and precision within a range of 6°–74°. In this reference method, only one Tx channel was active at a time, and the measurement was repeated eight times for each orientation to acquire data from all Tx channels. The number of cycles in the 2D B1‐MRF sequence was increased from 8 in the original implementation to 13, as the original setup was optimized for breath‐hold scanning, and artifacts were observed in the resulting maps when only 8 cycles were used during free‐breathing.

Acquisition parameters are listed in Table 2. The 2D B1‐MRF reference was acquired with a maximum nominal FA of 60°, while the hybrid 3D B1‐MRF had to be reduced to a maximum nominal FA of 55° to ensure that the 6‐min SAR limit was not exceeded. For comparison purposes, the 2D B1‐MRF reference maps were scaled by a factor of 55/60. The 2D B1‐MRF reference was acquired with a 2 × 2 mm^2^ resolution in transversal, coronal, and sagittal orientations. The 3D implementation had a resolution of 2 × 2 × 6 mm^3^. To facilitate comparisons along the head–feet dimension, which had a resolution of 6 mm in the 3D implementation, the 2D reference with a 2 mm resolution was averaged over 3 pixels in that dimension to match the resolution of the 3D hybrid implementation.

In contrast to classical B_1_^+^ mapping approaches, the FA in the MRF‐based B_1_^+^ mapping sequence is not constant but varies from 0° to a maximum FA over the FA pattern. For simplicity, the results section presents the maximum actual FA achieved during the MRF FA pattern [18] in the center slice, corresponding to a nominal FA of 55°.

The RF reference voltage was set to the system's maximum value of 478 V (for eight Tx channels combined) for all acquisitions. A conservative power‐controlled safety monitoring mode in the normal IEC mode was employed, limiting the RF power per Tx channel [22]. These power limits correspond to worst‐case 10 g averaged local SAR values in accordance with IEC guidelines. The 3D hybrid sequence reached 43% of the 10‐s limit and 77% of the 6‐min limit, while the 2D reference, with only one Tx channel active, reached 7% of the 10‐s limit and 12% of the 6‐min limit.

## 3 Results

**FIGURE 4:**
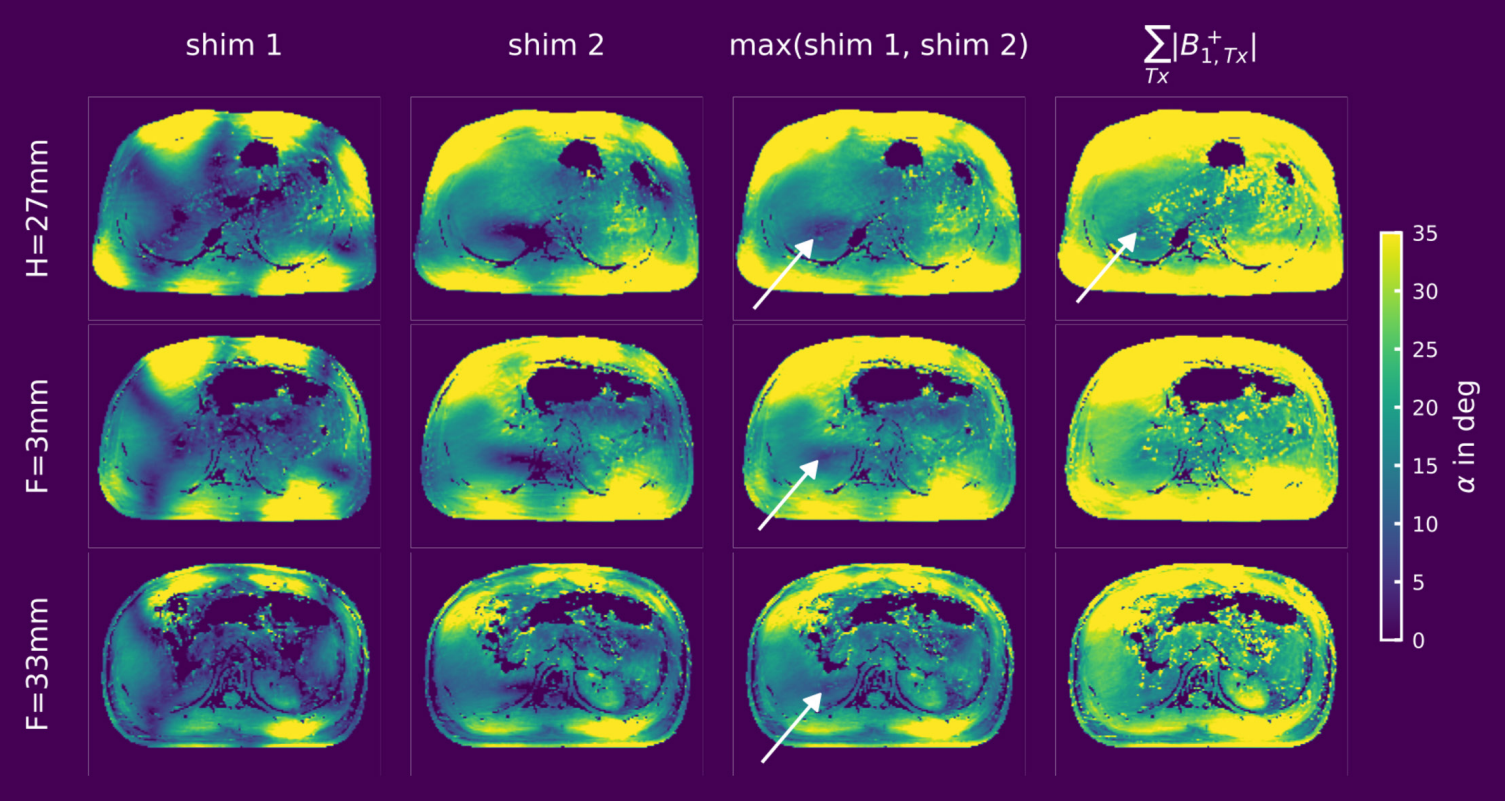
The first two columns display the two AMORE shims for three slices from the 3D B1‐MRF dataset at different positions along the head‐feet (HF) direction for one subject with a BMI of 25.9 kg/m^2^. The third column shows the maximum FA achieved over both shims, similar to the AMORE cost function. As highlighted by the arrows, even with two optimized shims, central regions of the liver experience FAs below 20° when using the 8 x 1 kW system and the body coil with a SINC pulse (TBWP of 4, pulse duration 1.33 ms). The last column presents the sum of magnitudes (SOM) B_1_^+^ maps, obtained by summing the magnitude of the channel‐wise absolute map $\left(\sum_{\mathrm{Tx}}|B_{1,\mathrm{Tx}}^{+}|\right)$. This represents the optimal scenario, which could only be experimentally achieved by acquiring multiple absolute B_1_^+^ maps. Even in this optimal scenario, achieving FAs greater than 20° for slices farther from the isocenter remains challenging.

Figure 4 presents the Tx channel‐combined absolute MRF‐based B_1_^+^ maps for three representative axial slices of the 3D dataset. The first two columns display the maps as reconstructed after the acquisition, while the third column shows the maximum FA across both shims (as used in the AMORE cost function in Equation 1). The fourth column indicates the FA achievable if the phases of each Tx channel align in each voxel ($\sum_{\mathrm{Tx}}|B_{1,\mathrm{Tx}}^{+}|$), referred to as the sum of magnitudes (SOM) of B_1_^+^ in this context. SOM represents the optimal scenario, which could only be experimentally achieved by acquiring multiple absolute B_1_^+^ maps, with the required shims equal to the number of voxels in the region of interest (ROI). As indicated by the arrows in the third and fourth rows, even with two or more different RF shims, there are regions in the liver where FAs > 20° cannot be achieved using the 8 × 1 kW amplifiers and the body coil with a SINC pulse with a TBWP = 4 and a pulse duration of 1.33 ms.

**FIGURE 5:**
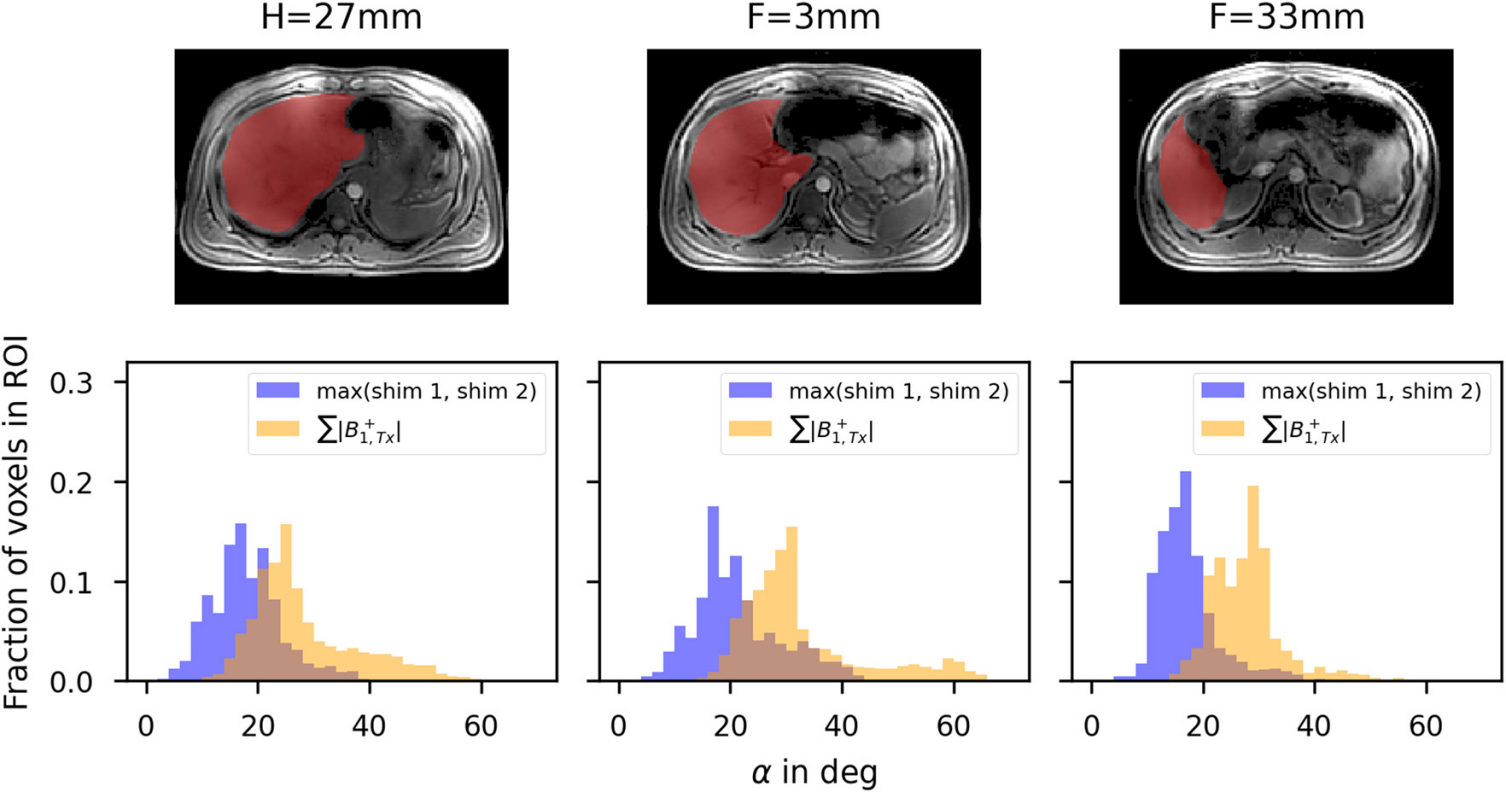
Histogram of Figure 4 for a ROI drawn on the liver (red shaded area in the first row). Across all three slices, the mean FA is 19.2° for the maximum over the two shims and 29.9° for the B_1_^+^ SOM. B_1_^+^ values change along the head‐feet (HF) direction, with the mean FA over the two shims decreasing in slices farther from the isocenter: 21.4° at F = 3 mm, 18.0° at H = 27 mm, and 17.2° at F = 33 mm. The same trend applies to the B_1_^+^ SOM, with values of 32.7° at F = 3 mm, 28.4° at H = 27 mm, and 28° at F = 33 mm.

This is evaluated in more detail in Figure 5, which shows the histogram of the FAs for the ROI covering the liver, corresponding to columns 3 and 4 of Figure 4. Across all voxels of all three slices, the mean FA is 19.2° for the maximum over the two shims and 29.9° for the B_1_^+^ SOM. Note that the slab profile is inherently corrected for in this approach and that the actual B_1_^+^ changes along the head‐feet (HF) direction. In the individual slices, the mean FA over the two shims decreases in slices farther from the isocenter along the HF direction, with 18.0° and 17.2° at H = 27 mm and F = 33 mm, respectively, compared to 21.4° at F = 3 mm. The same trend applies to the B_1_^+^ SOM, showing 28.4° and 28° at H = 27 mm and F = 33 mm, respectively, compared to 32.7° at F = 3 mm.

Supporting Information Figure S1 compares the absolute B_1_^+^ maps obtained axially in the isocenter using the proposed approach to those obtained using a vendor‐implemented 2D satTFL [13] method and a 3D radial phase encoded (RPE) actual flip angle imaging (AFI) [40] method. All methods were acquired using the same two AMORE shims at the RF amplifier or SAR limit. Accurate results were defined as deviations from a reference with |mean±SD| lower than 10%. For B1‐MRF, the minimum required FA was determined to be 6° [18], while satTFL requires FAs > 30° and AFI requires FAs > 45° [16]. Evaluating the percentage of data points in a liver ROI, where the maximum of the two shims is considered, 0% of points fall below the threshold for B1‐MRF, 49% for satTFL, and 95% for RPE‐AFI.

**FIGURE 6:**
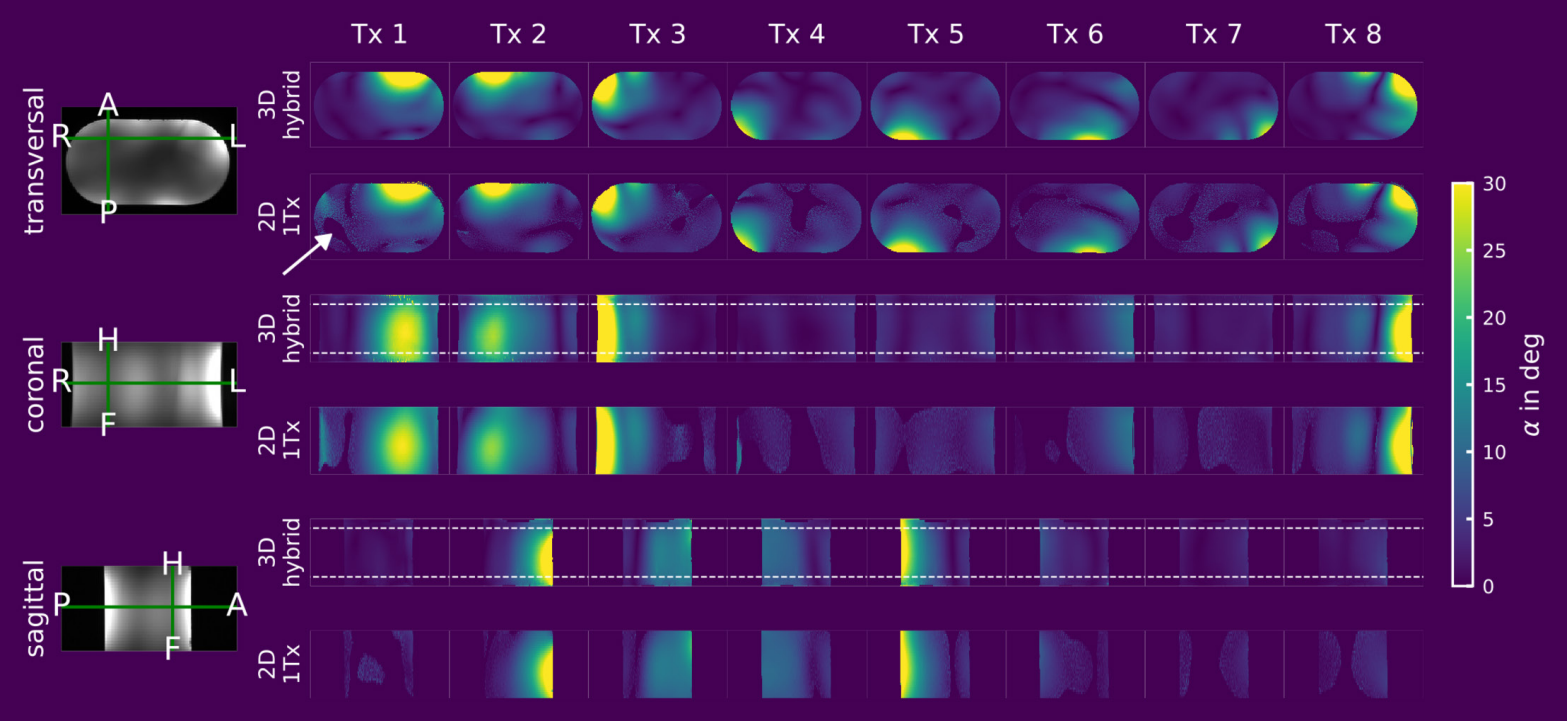
3D channel‐wise hybrid absolute B_1_^+^ maps for the PVP phantom. A transversal, coronal, and sagittal slice of the 3D hybrid dataset are shown alongside the same slices acquired with the 2D B1‐MRF sequence using only one Tx at a time. The white dotted line in the 3D measurements indicates the nominal slab thickness. The white arrow in the transversal orientation for the 2D measurement at Tx 1 marks a region where B_1_^+^ quantification was inaccurate due to low FA/SNR. This issue is not observed in the 3D hybrid implementation.

**FIGURE 7:**
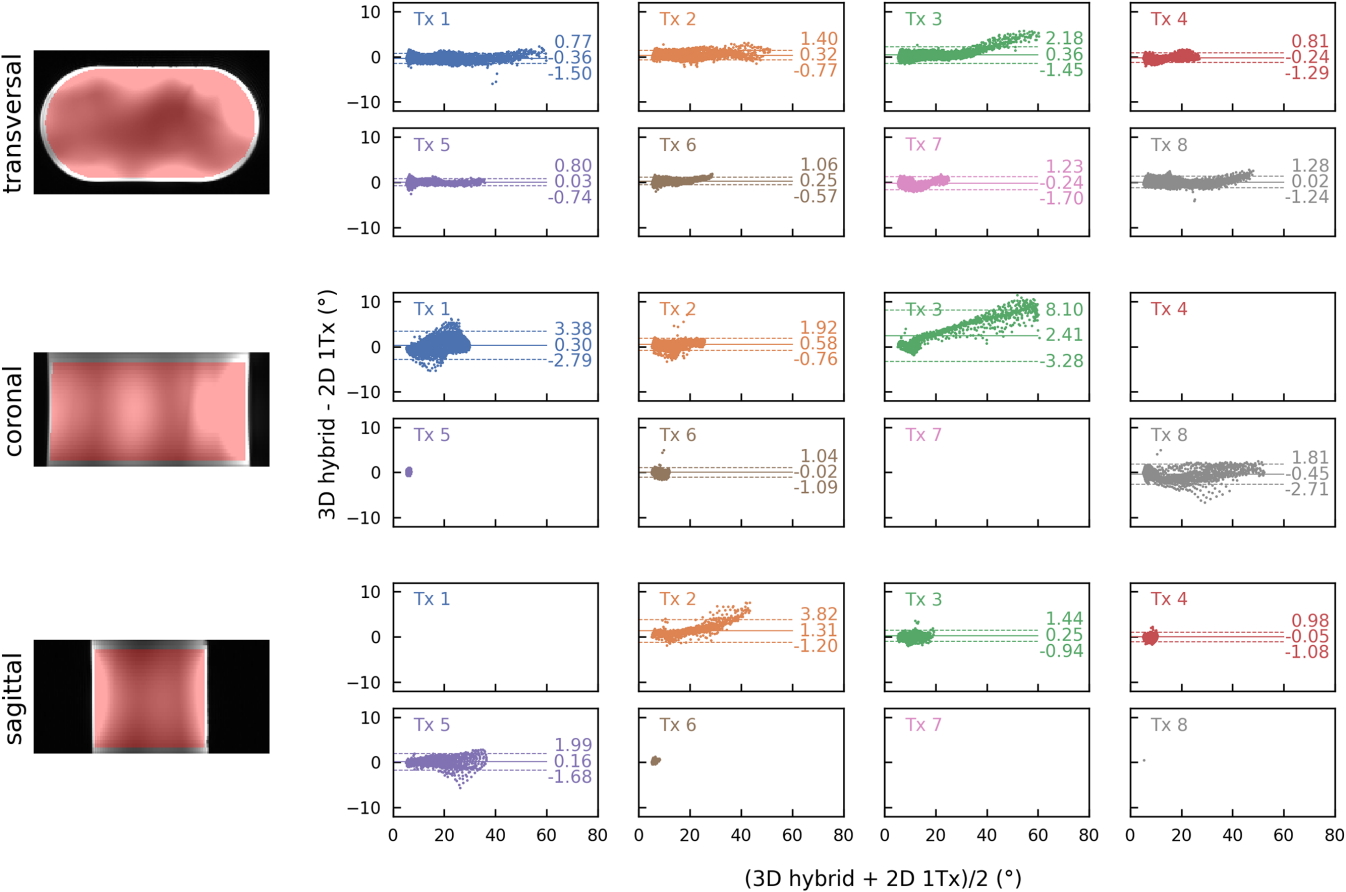
Quantitative comparison of the 3D hybrid approach to the 2D 1Tx measurement in a Bland–Altman plot for the phantom scan from Figure 6. All data points within the mask (red shaded areas on the left) and with FAs > 5° in both measurements were evaluated. As a result, some plots are empty, as only FAs below this threshold were observed for those Tx channels. For channels with more than 250 evaluated data points, the mean value is shown and visualized by a solid line, while the ±1.96 SDs from the mean value are shown with dotted lines. Across all evaluated data points, the overall mean ± SD of the difference was 0.19 ± 1.09°. For the different orientations and all data points across the different Tx channels, the mean ± SD of the differences were: 0.03 ± 0.67° for the transversal view, 0.59 ± 1.83° for the coronal view, and 0.41 ± 1.00° for the sagittal view.

**FIGURE 8:**
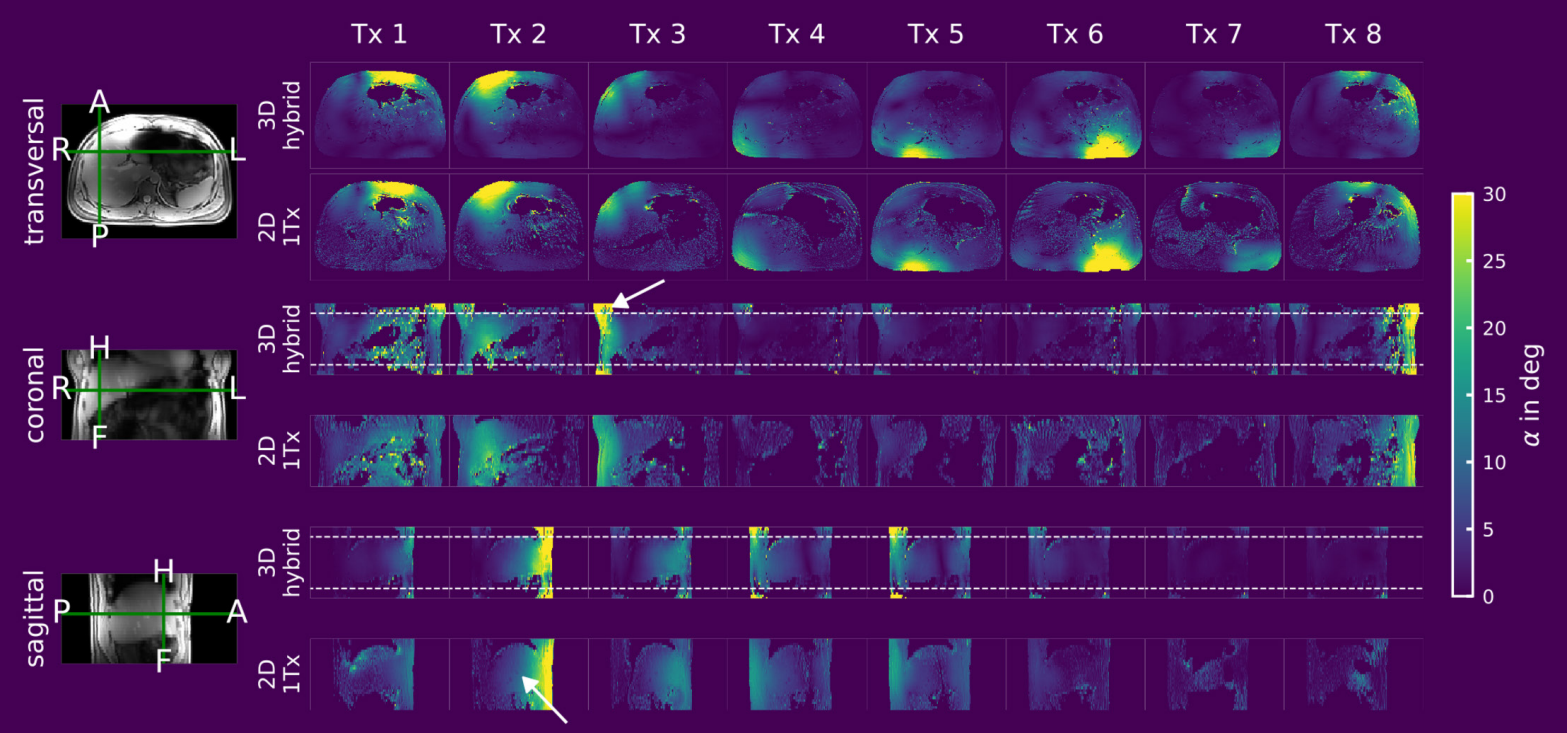
3D channel‐wise hybrid absolute B_1_^+^ maps for an in vivo scan of a subject with a BMI of 25.9 kg/m^2^. A transversal, coronal, and sagittal slice of the 3D hybrid dataset are shown alongside the same slices acquired with the 2D B1‐MRF sequence using only one Tx at a time. The white dotted line in the 3D measurements indicates the nominal slab thickness. In the coronal orientation for the 3D measurement at Tx 3, the white arrow marks a region where B_1_^+^ quantification appears inaccurate due to low signal from the slab profile. In the sagittal 2D 1Tx measurement, flow effects are visible in Tx 2, which are not present in the 3D hybrid implementation.

**FIGURE 9:**
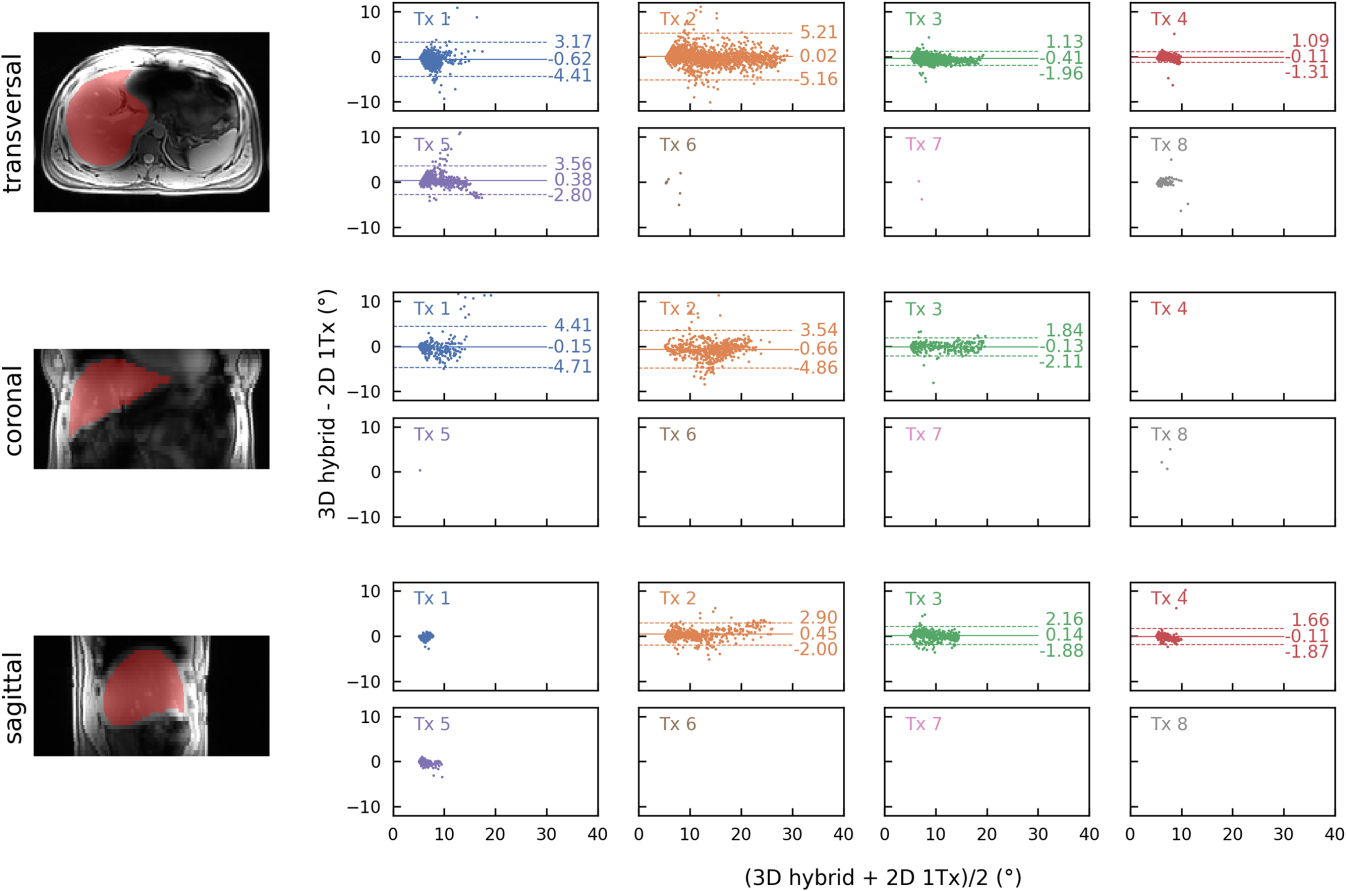
Quantitative comparison of the 3D hybrid approach to the 2D 1Tx measurement in a Bland–Altman plot for the subject with a BMI of 25.9 kg/m^2^ from Figure 8. All data points within the mask (red shaded areas on the left) and with FAs > 5° in both measurements were evaluated. As a result, some plots are empty, as only FAs below this threshold were observed for those Tx channels. For channels with more than 250 evaluated data points, the mean value is shown and visualized by a solid line, while the ± 1.96 SDs from the mean value are shown with dotted lines. Across all evaluated data points, the overall mean ± SD of the difference was −0.12 ± 1.85°. For the different orientations and all data points across the different Tx channels, the mean ± SD of the differences were −0.13 ± 1.99° for the transversal view, −0.40 ± 1.99° for the coronal view, and 0.14 ± 1.05° for the sagittal view.

Figures 6, 7, 8, and 9 compare the channel‐wise B_1_^+^ maps of the 3D hybrid approach to the 2D reference for both the phantom and in vivo scans. Figure 6 shows the FA maps obtained in the PVP phantom for coronal, transversal, and sagittal slices using the 3D hybrid approach, compared to the corresponding 2D slices acquired with the 2D B1‐MRF sequence, where only a single Tx channel was active. Visually, there is high agreement between the two measurements across all orientations. Although B1‐MRF exhibits a large dynamic range, the 2D 1Tx reference reveals areas of inconsistent B_1_^+^ values in low FA and SNR regions, which are partially masked due to the low signal (marked by an arrow in the transversal 2D 1Tx slice). The dotted white line in the 3D hybrid images indicates the nominal slab thickness, shown with coronal and sagittal slices. In the oversampling region outside the nominal slab, artifacts are perceptible, particularly in the outermost slices.

A quantitative comparison of the two measurements is shown in Bland–Altman plots in Figure 7. The contrast image on the left shows the ROI used for quantitative comparison, where all points with α > 5° within the ROI were evaluated. The subplots also include the mean values and ±1.96 SDs for all comparisons where more than 250 data points (*n* > 250) were available. Overall, there is strong quantitative agreement between the two scans, with a mean ± SD difference of 0.19 ± 1.09° when considering all data points within the mask (Tx 1–8 and all orientations). Comparing the different orientations for all data points across the different Tx channels with n > 250, the highest agreement is observed in the transversal view, with a mean ± SD of 0.03 ± 0.67°. In the coronal view, the mean ± SD difference is 0.59 ± 1.83°, while in the sagittal view, it is 0.41 ± 1.00°. Supporting Information Figures S3 and S4 investigate the reproducibility of the phantom measurements. Three scans were performed with a 2 h interval between the start of each acquisition. When evaluating all data points simultaneously, a mean ± SD difference of 0.03 ± 0.67° was observed between the first and the last scan.

Figure 8 is structured similarly to Figure 6 and shows the results of an in vivo scan of one subject with an BMI of 25.9 kg/m^2^. Visually, there is high agreement between the two scans, although the 2D 1Tx scans exhibit inconsistent values in low SNR regions. Deviations are observed in regions near the coil elements, specifically in slices close to the edge of the excited slab, such as in the coronal view of Tx 3 (marked by an arrow). In the sagittal view, artifacts caused by flow are observed in the 2D 1Tx scans, which are not observed in the 3D hybrid scan (marked by another arrow).

A quantitative comparison for the in vivo scan is shown in Bland–Altman plots in Figure 9 (similar to Figure 7 for the phantom). An ROI covering the liver was manually drawn for evaluation (c.f. contrast image in Figure 9). Overall, there is a quantitative agreement between the two scans, with a mean ± SD difference of −0.12 ± 1.85° when considering all data points. Comparing the different orientations for all data points across the different Tx channels with *n* > 250, the highest agreement is observed in the sagittal view, with a mean ± SD of 0.14 ± 1.05°. In the transversal view, the mean ± SD difference is 0.13 ± 1.99°, while in the coronal view, it is −0.40 ± 1.99°.

Supporting Information Figures S4–S7 present similar plots for the other two subjects with BMIs of 27.8 kg/m^2^ and 18.1 kg/m^2^. For the subject with the higher BMI (Supporting Information Figures S4 and S5), a lower agreement is observed between the 3D hybrid scan and the 2D references, with a mean ± SD difference of −0.83 ± 3.20° for all data points. In contrast, for the subject with the lower BMI (Supporting Information Figure S6 and S7), a higher agreement is observed between the 3D hybrid scan and the 2D references, with a mean ± SD difference of −0.58 ± 1.97° across all data points. More details are provided in the Supporting Information Figures.

## 4 Discussion

In this work, a 3D channel‐wise hybrid B_1_^+^ mapping approach based on B1‐MRF was developed and validated. Phantom and in vivo scans demonstrated strong agreement with the 2D reference measurement, where only one Tx channel was active at a time. This comparison was enabled by the high accuracy of MRF‐based B_1_^+^ mapping at low FAs [18], which remains a challenge for other established UHF B_1_^+^ mapping methods, particularly in vivo.

So far, only a few other methods have been implemented for channel‐wise B_1_^+^ mapping in the human body at UHF that are listed in Table 1. Runderkamp et al. [9] used an interferometric approach based on 2D multislice DREAM [12]. While homogeneous GRE images were obtained using RF pulses based on kt‐points calculated from these maps, no validation of the method's accuracy was provided. Biases in the DREAM method are expected in regions of flow [41], as stated by the authors, and, like other approaches, in low FA and low SNR regions [42].

Brunheim et al. [10] proposed B1TIAMO, which introduced two shims for absolute B_1_^+^ mapping in a hybrid approach, a technique also employed in this work. However, compared to our 8 × 1 kW system, B1TIAMO results were obtained using a system with 32 × 0.7 kW total power, albeit the power was used to drive a remote and not a local coil array. This configuration enabled the achievement of FAs of 40° at the body center using a CP^+^ mode with satTFL to obtain accurate absolute B_1_^+^ maps, a result that would be difficult to achieve with the lower power of our system.

Kent et al. [11] used a hybrid strategy similar to B1TIAMO, but achieved 3D volume acquisition in the heart within a single breath‐hold by using a smaller matrix size of 24^3^ and transmit low rank (TxLR) [43] for accelerated acquisition of the channel‐wise GRE images. The reported dynamic for the underlying sandwiched satTFL [14] method spans from 40° to 120° [17]. Simulations showed that acquiring only the central k‐space part, resulting in a resolution of 15.8 × 15.8 × 11.9 mm^3^, had minimal impact on map accuracy. To the best of our knowledge, this is presently the only other unbiased 3D encoding technique for UHF B_1_^+^ mapping in the body, provided that sufficiently high FAs can be achieved.

Despite these methods, no gold standard or an established reference method with high accuracy exists for UHF B_1_^+^ mapping in the body, primarily due to the limited dynamic range of available methods and the inherent limitations of each technique. Given that a reference method must offer high sensitivity across the expected FA range and that free‐breathing measurements are advantageous for body imaging, we believe that MRF‐based B_1_^+^ measurements represent a significant step toward addressing this gap.

A key challenge of UHF B_1_^+^ mapping in the body is the limited transmit efficiency, i.e., B_1_^+^ per applied RF voltage, of body arrays compared to head arrays. Achieving FAs > 20° with the applied SINC‐shaped pulses in central body regions remains challenging, especially with systems that have limited RF power, even when using optimized shims (see Figures 4 and 5). This challenge is particularly relevant for existing methods, as biased results are expected for FAs below approximately 30° [16, 17, 18]. Even when considering a block‐shaped RF pulse, as used in satTFL [13] and AFI [15] with a 0.5 ms duration instead of the sinc‐shaped pulse used here, the results from a slice in the isocenter (shown in Supporting Information Figure S1) demonstrate that 49–95% of the B_1_^+^ maps in the liver fall below the threshold where the data is considered accurate. In contrast, for B1‐MRF, 0% of data points fall below this range with the applied SINC‐shaped pulse and when running at the power limit, significantly reducing the issue.

Besides the general high qualitative agreement between the channel‐wise comparisons of the 3D hybrid approach and the 2D reference measurements, some quantitative deviations were observed, which have several causes. Higher quantitative agreement was found for the phantom scan compared to the in vivo scan, with subject motion potentially contributing to this difference. In general, the 2D reference is expected to be accurate for regions with sufficient FA (> 6°) and SNR. However, artifacts are observed in regions farther from the coil elements, where low FAs occur. These artifacts arise from the noise‐dominated signal in these regions, causing the MRF reconstruction to match noise instead of the actual signal, leading to incorrect B_1_^+^ values. Since these regions were included in the quantitative comparison as long as the matched FA was > 5°, a bias in metrics such as mean and SD is expected, as FA values above this threshold are also observed in noise‐dominated regions. In general, since the B_1_^+^ maps were acquired with all Tx channels activated, leading to higher SNR, the 3D method provides more accurate results in these regions.

In addition, deviations between the 3D hybrid approach and the 2D reference are possible, as the exact position of the excited slice is influenced by B_0_ variations. Additional ΔB_0_ measurements were conducted to estimate the ΔB_0_ range, which was found to be within ±300 Hz across the liver. Slice shifts of up to 0.5 mm are expected for the 2D reference measurements within this ΔB_0_ range. In the 3D case, such a ΔB_0_ offset can cause a shift of the slab excitation profile of up to 12 mm in the worst case, potentially leading to aliasing effects in the lowest/highest slices. Furthermore, the shifted slab profile can introduce a minor mismatch between the measured signal and the signal from the dictionary used during MRF matching, which could be fully mitigated by including a ΔB_0_ map.

The impact of subject motion was investigated in Supporting Information Figures S8–S10, where twice the amount of data was acquired, and the data was binned retrospectively into motion states using a motion surrogate derived from the central k‐space line along the z‐dimension, similar to XD‐GRASP [44]. Only minor spatial variations between the exhale and averaged motion states were observed in the contrast images (c.f. Supporting Information Figure S8). A comparison of the resulting B_1_^+^ maps, including data from all motion states combined versus the exhale‐only motion states, revealed only minor B_1_^+^ deviations (c.f. Supporting Information Figures S9 and S10) with mean ± SD difference of 0.33 ± 1.45° between the exhale and averaged motion states in the liver. These results are consistent with those of Riel et al. [32], where good agreement was found between static and moving scans when the k_z_ dimension was sampled first, as applied in this work.

Furthermore, while the 3D B_1_^+^ maps are inherently corrected for the slab profile through the MRF dictionary, Supporting Information Figure S11 highlights the importance of including the slab profile in the dictionary simulations. Without this inclusion, errors increase with increasing distance from the center of the slab. However, the actual signal decreases along the slab selection direction with distance from the slab center due to profile variations, as shown in Figure 3. This results in decreasing SNR and thus deviations near or beyond the edge of the slab, as observed for some channels in the in vivo scans (e.g., Figure 8: Tx 3 coronal). Although the deviations are relatively minor, this issue could be mitigated by using RF pulses with a higher TBWP, at the cost of increased power demands to achieve the same FA in the center of the slab.

The basic assumption of the hybrid combination is that the intensity of the 3D channel‐wise GRE images scales linearly with the FA [45], which holds true when i) the FA of the GRE images is low (i.e., sin(α) ≈ α) and ii) the signal is not saturated (i.e., the FA is smaller than the Ernst angle). If these assumptions were not fulfilled, non‐linearities at higher FAs would be expected in the correlation plots shown in Figure 7 and 9. However, no such non‐linearities are apparent. Since the acquisition order of the individual Tx channels is cycled during the acquisition, saturation effects are reduced by the increased effective TR for voxels near the coil elements [10].

A general advantage of 3D B_1_^+^ mapping approaches, compared to most 2D methods, is the higher accuracy in regions with flow. For example, DREAM [12] is known to be flow sensitive due to the STEAM preparation [41]. In the case of B1‐MRF, flow artifacts can be observed in the in vivo maps of vascular areas with the 2D implementation (also c.f. Supporting Information Figure S4). In contrast, such artifacts are less or not evident in the 3D hybrid implementation.

While the presented method offers higher accuracy in low FA regions and higher resolution compared to other available B_1_^+^ mapping methods for body imaging at UHF, the method comes at the cost of significantly longer acquisition times, currently totaling 9 min 36 s (3 min 48 s for channel‐wise GRE images and 5 min 48 s for the two absolute MRF‐based B_1_^+^ maps). This makes the method in its current implementation unlikely to be used for subject‐tailored RF pulse design calibration within a single scan session, as existing methods offer notable time benefits, with channel‐wise maps being acquired in a single breath‐hold [10, 11].

Nonetheless, there are important scenarios where acquisition time is less critical and accuracy and detail are prioritized. This includes validating faster B_1_^+^ approaches to assess their level of accuracy in vivo. Additionally, a critical application is the safety validation of electromagnetic (EM) simulations, where accurate B_1_^+^ reference maps are essential to validate the simulation results. EM simulations are typically performed with resolutions ≤ 2 mm [19, 20, 21, 22], as increases in the maximum 10 g SAR of up to 97% have been observed when the model resolution is increased from 5 to 2 mm [46]. To match the simulation resolution and enable meaningful validation, experimentally acquired B_1_^+^ maps are also typically obtained with in‐plane resolutions ≤ 2 mm [19, 20, 21, 22]. Another application is electrical properties tomography (EPT), where high‐resolution B_1_^+^ maps are needed, as the resolution of these maps directly affects the resolution of the resulting quantitative maps [47]. In this context, multichannel B_1_^+^ maps are essential, e.g., for gEPT [7, 8]. Moreover, the technique would strongly benefit applications that rely on accurate B_1_^+^ libraries from different subjects. One example is the design of universal pulses [23, 24], which optimize subject‐independent RF pulses based on B_1_^+^ maps from a library of various subjects, eliminating the need for time‐consuming calibration. Another example is the training of AI‐based approaches to estimate channel‐wise B_1_^+^ maps from a quick calibration or localizer scan using neural networks [25].

Still, if both fast acquisition and high accuracy is needed, several strategies could be employed to shorten the scan time of the presented technique. Supporting Information Figure S12 illustrates the trade‐off between acquisition time and resolution, where a dataset with a 6 mm isotropic resolution was acquired in 6 min 11 s, and a dataset with a 10 mm isotropic resolution was acquired in 2 min 30 s. Additional measures could further reduce acquisition time while maintaining a 2 × 2 × 6 mm^3^ resolution. First, undersampling along the partition dimension using variable density sampling [48] could be implemented. For example, further acceleration could be achieved by sampling a larger portion of k‐space in one TR by switching from a stack‐of‐stars to a stack‐of‐spirals acquisition. Channel‐wise image acquisition could be sped up by exploiting low‐rank conditions between channels, such as using TxLR [43] for Cartesian images. The duration of the absolute mapping could also be reduced by optimizing the MRF FA pattern using the Cramér‐Rao bound [32, 49, 50, 51] to increase sensitivity.

## 5 Conclusion

Free‐breathing, high‐resolution 3D Tx multi‐channel hybrid B_1_^+^ mapping based on B1‐MRF was demonstrated in the human body at 7 T in under 10 min of acquisition time. Compared to classical approaches, it offers improved accuracy, particularly in low FA regions. This method could represent a significant step toward establishing a Tx channel‐wise reference B_1_^+^ mapping technique for body imaging at 7 T and beyond.

## Supporting information


**Figure S1** Comparison of the absolute B_1_^+^ map to satTFL and RPE‐AFI for a transversal slice at the isocenter in a subject with a BMI of 25.9 kg/m^2^. (A) The resulting FA maps normalized by the nominal FA (B1‐MRF: 55°; satTFL: 90°; RPE‐AFI: 80°). For the hybrid combination, it is crucial that the max (shim 1, shim 2) map does not exhibit dropout areas, as this would hinder accurate FA quantification. In contrast, for satTFL and RPE‐AFI, inconsistencies (marked by white arrows) are visible, which prevent accurate FA quantification in these regions. These inconsistencies are not observed in the B1‐MRF approach. (B) The histogram of the actual FA of the max (shim 1, shim 2) maps from panel (A) within a region of interest (ROI) drawn on the liver, as shown on the left. The dotted line represents the lower threshold for accurate results, defined as deviations from a reference evaluated as|mean ± SD|lower than 10%. For B1‐MRF, this threshold was experimentally determined to be 6° (Lutz et al.^22^), while for satTFL, FAs > 30° and for AFI, FAs > 45° are required (Pohmann et al.^20^). Evaluating the percentage of data points falling below the respective threshold, 0% of points fall below for B1‐MRF, 49% for satTFL, and 95% for RPE‐AFI. Acquisition parameters of the reference sequences were as follows: satTFL: pulse type: RECT; pulse duration, 0.5 ms; nominal FA, 90° (played out at amplifier limit of 169 V per Tx channel), TR = 5000 ms, TE = 2.2 ms, slice thickness = 5 mm, FOV = 384 x 264 mm^2^, resolution = 4 × 4 mm^2^, TA per shim = 10 s. RPE‐AFI: pulse type, RECT; pulse duration, 0.5 ms; nominal FA, 80° (close to SAR limit); TR1/TR2 = 10/50 ms, FOV = 320 × 320 × 256 mm^3^, resolution = 4 × 4 × 4 mm^3^, TA per shim = 6 min 9 s.
**Figure S2** Qualitative results of phantom reproducibility measurements using the same phantom as in Figure 6. Three consecutive scans were performed, with a 2 h interval between the start of each acquisition. Before each scan, the center frequency was re‐adjusted; no further calibration was applied.
**Figure S3** Quantitative evaluation of the reproducibility measurements from Supporting Information Figure S2, comparing the first and last scan. All data points within the mask (red shaded areas on the left) and with FAs > 5° in both measurements were evaluated. For channels with more than 250 evaluated data points, the mean value is shown and visualized by a solid line, while the ±1.96 SDs from the mean value are shown with dotted lines. Across all evaluated data points, the overall mean±SD of the difference was −0.02 ± 0.49°. For the different orientations and all data points across the different Tx channels, the mean±SD of the differences were 0.02 ± 0.44° for the transversal view, −0.05 ± 0.60° for the coronal view, and 0.19 ± 0.56° for the sagittal view.
**Figure S4** 3D channel‐wise hybrid absolute B_1_^+^ maps for an in vivo scan of a subject with a BMI of 27.8 kg/m^2^. A transversal, coronal, and sagittal slice of the 3D hybrid dataset are shown, alongside the same slices acquired with the 2D B1‐MRF sequence using only one Tx at a time. The white dotted line in the 3D measurements indicates the nominal slab thickness.
**Figure S5** Quantitative comparison of the 3D hybrid approach to the 2D 1Tx measurement in a Bland–Altman plot for the subject with a BMI of 27.8 kg/m^2^ from Supporting Information Figure S4. All data points within the mask (red shaded areas on the left) and with FAs > 5° in both measurements were evaluated. For channels with more than 250 evaluated data points, the mean value is shown and visualized by a solid line, while the ±1.96 SDs from the mean value are shown with dotted lines. Across all evaluated data points, the overall mean±SD of the difference was −0.83 ± 3.20°. For the different orientations and all data points across the different Tx channels, the mean±SD of the differences were −1.05 ± 3.40° for the transversal view, −0.47 ± 2.57° for the coronal view, and 0.02 ± 2.27° for the sagittal view.
**Figure S6** 3D channel‐wise hybrid absolute B_1_^+^ maps for an in vivo scan of a subject with a BMI of 18.1 kg/m^2^. A transversal, coronal, and sagittal slice of the 3D hybrid dataset are shown, alongside the same slices acquired with the 2D B1‐MRF sequence using only one Tx at a time. The white dotted line in the 3D measurements indicates the nominal slab thickness. In the transversal 2D 1Tx measurement, flow effects are visible in the maps of the single Tx channels, which are not present in the 3D hybrid implementation.
**Figure S7** Quantitative comparison of the 3D hybrid approach to the 2D 1Tx measurement in a Bland–Altman plot for the subject with a BMI of 18.1 kg/m^2^ from Supporting Information Figure S6. All data points within the mask (red shaded areas on the left) and with FAs > 5° in both measurements were evaluated. For channels with more than 250 evaluated data points, the mean value is shown and visualized by a solid line, while the ±1.96 SDs from the mean value are shown with dotted lines. Across all evaluated data points, the overall mean±SD of the difference was −0.58 ± 1.97°. For the different orientations and all data points across the different Tx channels, the mean±SD of the differences were −0.05 ± 1.63° for the transversal view, −0.78 ± 1.97° for the coronal view, and −0.45 ± 2.28° for the sagittal view.
**Figure S8** Illustration of the motion‐resolved reconstruction. Similar to XD‐GRASP^45^, the data is binned into different motion states. (A) A 1D FFT is performed along the k_z_ dimension for *k*_*x*_ = *k*_*y*_ = 0 for each set. The data is then concatenated into a 2D matrix, where the first dimension represents time and the second dimension represents z + coil. Unlike qualitative MRI, the data at each time point is normalized by its maximum value to suppress contrast changes induced by the applied FA pattern. The red dotted line indicates the start of the next cycle and demonstrates that the signal variations in Rx 4 do not follow the periodicity of the MRF FA pattern. Principal component (PC) analysis was applied to the 2D matrix shown in (A), and the 2nd PC is displayed, as the 1st PC still shows the MRF FA pattern. The signal was low‐pass filtered, and the FFT spectrum reveals a peak at 0.44 Hz, which corresponds to the frequency range expected for respiratory motion. (C) The reconstruction of data from different motion states for a sagittal orientation at three different positions, marked in the transversal image on the left. Data from different sets and shims are combined, leading to mixed image contrast. In regions of the back, the motion between inhale and exhale shows a detectable effect at the upper border of the liver, while in the middle and front positions, nearly no effect from respiration is observed. Comparing the averaged state to the exhale state at the different coronal positions, the variation along the z‐dimension for the top of the liver is less than 1 pixel.
**Figure S9** Comparison of the exhale and averaged motion states for the 3D hybrid B1‐MRF approach. The acquired data was doubled compared to the parameters stated in Table 2, resulting in double the acquisition time. For the exhaled motion state, which accounted for 40% of the acquired data, the data was binned as shown in Supporting Information Figure S8, and only the data collected during exhalation was used for image reconstruction and MRF matching. In contrast, for the averaged motion state, all data was reconstructed as in the other data presented in this work. In this qualitative comparison, no substantial differences are observed between the averaged and exhaled motion states.
**Figure S10** Quantitative comparison of the exhale and averaged motion state in a Bland–Altman plot for the data shown in Supporting Information Figure S9. All data points within the mask (red shaded areas on the left) and with FAs > 5° in both measurements were evaluated. For channels with more than 250 evaluated data points, the mean value is shown and visualized by a solid line, while the ±1.96 SDs from the mean value are shown with dotted lines. Across all evaluated data points, the overall mean±SD of the difference was 0.33 ± 1.45°. For the different orientations and all data points across the different Tx channels, the mean±SD of the differences were 0.40 ± 1.44° for the transversal view, 0.18 ± 2.07° for the coronal view, and 0.19 ± 0.65° for the sagittal view.
**Figure S11** Comparison of including the slab profile in the signal model (w/ SP) versus not including it (w/o SP) for the phantom experiment from Figure 6. No observable difference in the B_1_^+^ results is found for the central slices between w/ SP and w/o SP, and both agree well with the 2D reference. However, in the coronal and sagittal views, not including the slab profile leads to differences that increase with the distance from the slab center. Compared to the 2D reference, substantial differences are observed when the slab profile is excluded in the 3D casefrom the signal model, which are not present when the slab profile is included. Quantitative evaluation within the ROIs drawn in Figure 7 shows that for the transversal orientation at the isocenter, the mean±SD difference to the 2D 1Tx reference remains constant (w/ SP and w/o SP: 0.03 ± 0.67°). However, for the coronal and sagittal orientations, the absolute mean difference increases by a factor greater than 5.0, and the standard deviation increases by a factor greater than 2.6 for the w/o SP reconstruction compared to the w/ SP reconstruction. Specifically, for the coronal view, the mean ± SD is w/ SP 0.59 ± 1.83° and w/o SP −2.97 ± 4.85°, while for the sagittal view, it is w/ SP 0.41 ± 1.00° and w/o SP −2.07 ± 3.55°.
**Figure S12** Flexibility of the hybrid 3D B1‐MRF method in vivo. The first row shows the transversal slice at the isocenter with a 2 x 2 x 6 mm^3^ resolution, acquired with a TA of 9 min 36 s, which was used throughout this work. The second row shows a 6 mm^3^ isotropic resolution, reducing the acquisition time to 6 min 11 s. The last row shows a 10 mm^3^ isotropic resolution, acquired in 2 min 30 s.

## Data Availability

The data that support the findings of this study are available from the corresponding author upon reasonable request.
