# Supplementary material for: Accurate MRF‐Based 3D Multi‐Channel B1 + Mapping in the Human Body at 7 T
Source: NMR Biomed. 2025 Jun 18;38(8):e70080. doi: 10.1002/nbm.70080 (PMC12176530; doi:10.1002/nbm.70080)
Supplement: Supplementary file 1 — Figure S1 Comparison of the absolute B1 + map to satTFL and RPE‐AFI for a transversal slice at the isocenter in a subject with a BMI of 25.9 kg/m2. (A) The resulting FA maps normalized by the nominal FA (B1‐MRF: 55°; satTFL: 90°; RPE‐AFI: 80°). For the hybrid combination, it is crucial that the max (shim 1, shim 2) map does not exhibit dropout areas, as this would hinder accurate FA quantification. In contrast, for satTFL and RPE‐AFI, inconsistencies (marked by white arrows) are visible, which prevent accurate FA quantification in these regions. These inconsistencies are not observed in the B1‐MRF approach. (B) The histogram of the actual FA of the max (shim 1, shim 2) maps from panel (A) within a region of interest (ROI) drawn on the liver, as shown on the left. The dotted line represents the lower threshold for accurate results, defined as deviations from a reference evaluated as|mean ± SD|lower than 10%. For B1‐MRF, this threshold was experimentally determined to be 6° (Lutz et al.22), while for satTFL, FAs > 30° and for AFI, FAs > 45° are required (Pohmann et al.20). Evaluating the percentage of data points falling below the respective threshold, 0% of points fall below for B1‐MRF, 49% for satTFL, and 95% for RPE‐AFI. Acquisition parameters of the reference sequences were as follows: satTFL: pulse type: RECT; pulse duration, 0.5 ms; nominal FA, 90° (played out at amplifier limit of 169 V per Tx channel), TR = 5000 ms, TE = 2.2 ms, slice thickness = 5 mm, FOV = 384 x 264 mm2, resolution = 4 × 4 mm2, TA per shim = 10 s. RPE‐AFI: pulse type, RECT; pulse duration, 0.5 ms; nominal FA, 80° (close to SAR limit); TR1/TR2 = 10/50 ms, FOV = 320 × 320 × 256 mm3, resolution = 4 × 4 × 4 mm3, TA per shim = 6 min 9 s. Figure S2 Qualitative results of phantom reproducibility measurements using the same phantom as in Figure 6. Three consecutive scans were performed, with a 2 h interval between the start of each acquisition. Before each scan, the center frequency was re‐adjusted; [file NBM-38-e70080-s001.docx]

# Supplementary Information

_
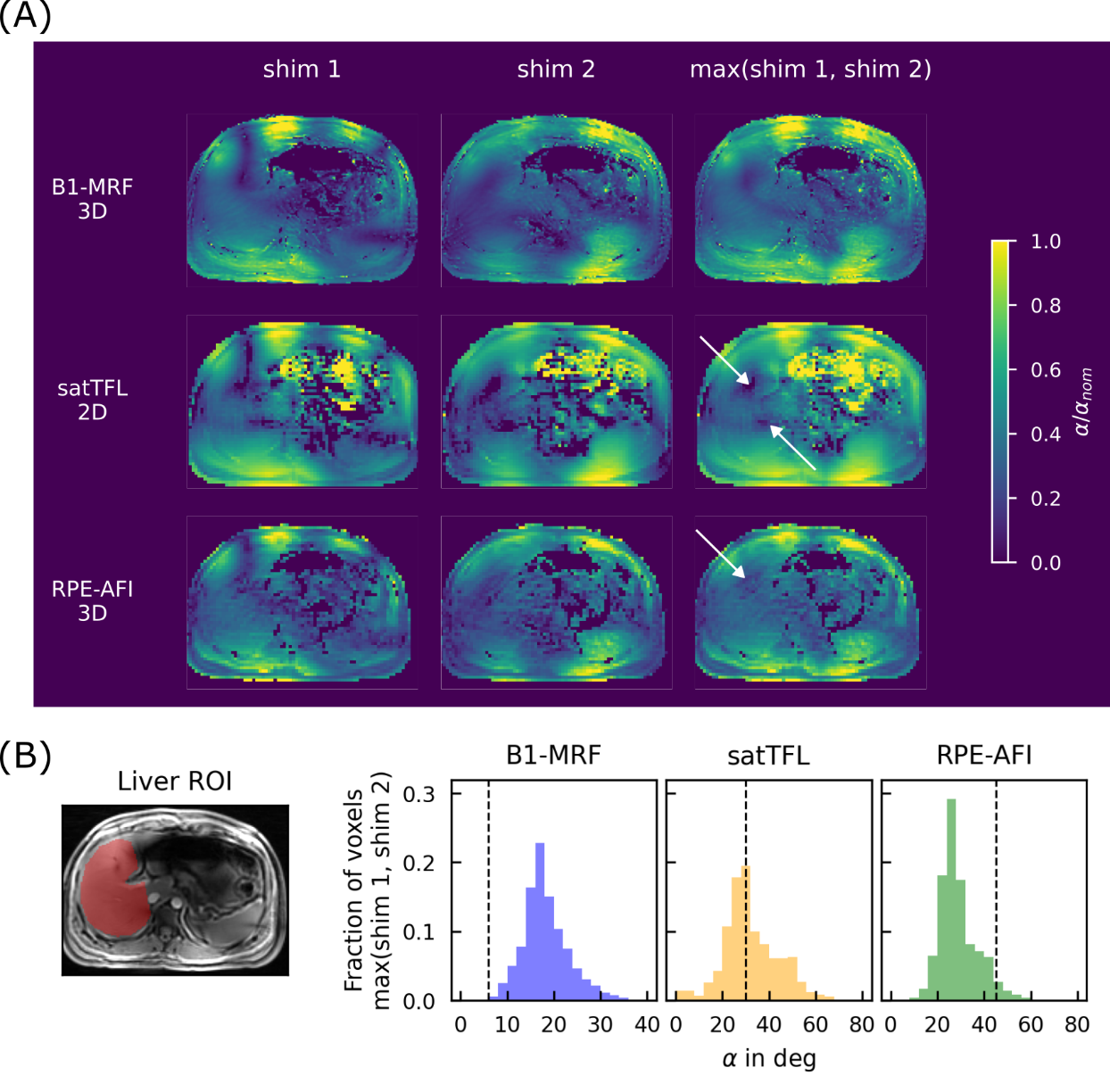
_

Supporting Information Figure S1: Comparison of the absolute B_1_^+^ map to satTFL and RPE-AFI for a transversal slice at the isocenter in a subject with a BMI of 25.9 kg/m². (A) shows the resulting FA maps normalized by the nominal FA (B1-MRF: 55°, satTFL: 90°, RPE-AFI: 80°). For the hybrid combination, it is crucial that the max(shim 1, shim 2) map does not exhibit dropout areas, as this would hinder accurate FA quantification. In contrast, for satTFL and RPE-AFI, inconsistencies (marked by white arrows) are visible, which prevent accurate FA quantification in these regions. These inconsistencies are not observed in the B1-MRF approach. (B) shows the histogram of the actual FA of the max(shim 1, shim 2) maps from panel (A) within a region of interest (ROI) drawn on the liver, as shown on the left. The dotted line represents the lower threshold for accurate results, defined as deviations from a reference evaluated as |mean ± SD| lower than 10%. For B1-MRF, this threshold was experimentally determined to be 6° (Lutz et al.^22^), while for satTFL, FAs > 30° and for AFI, FAs > 45° are required (Pohmann et al.^20^). Evaluating the percentage of data points falling below the respective threshold, 0% of points fall below for B1-MRF, 49% for satTFL, and 95% for RPE-AFI. Acquisition parameters of the reference sequences were as follows: satTFL: pulse type: RECT, pulse duration: 0.5 ms, nominal FA: 90° (played out at amplifier limit of 169V per Tx channel), TR = 5000 ms, TE = 2.2 ms, slice thickness = 5 mm, FOV = 384 x 264 mm², resolution = 4 x 4 mm², TA per shim = 10 s. RPE-AFI: pulse type: RECT, pulse duration: 0.5 ms, nominal FA: 80° (close to SAR limit), TR1/TR2 = 10/50 ms, FOV = 320 x 320 x 256 mm³, resolution = 4 x 4 x 4 mm³, TA per shim = 6 min 9 s.


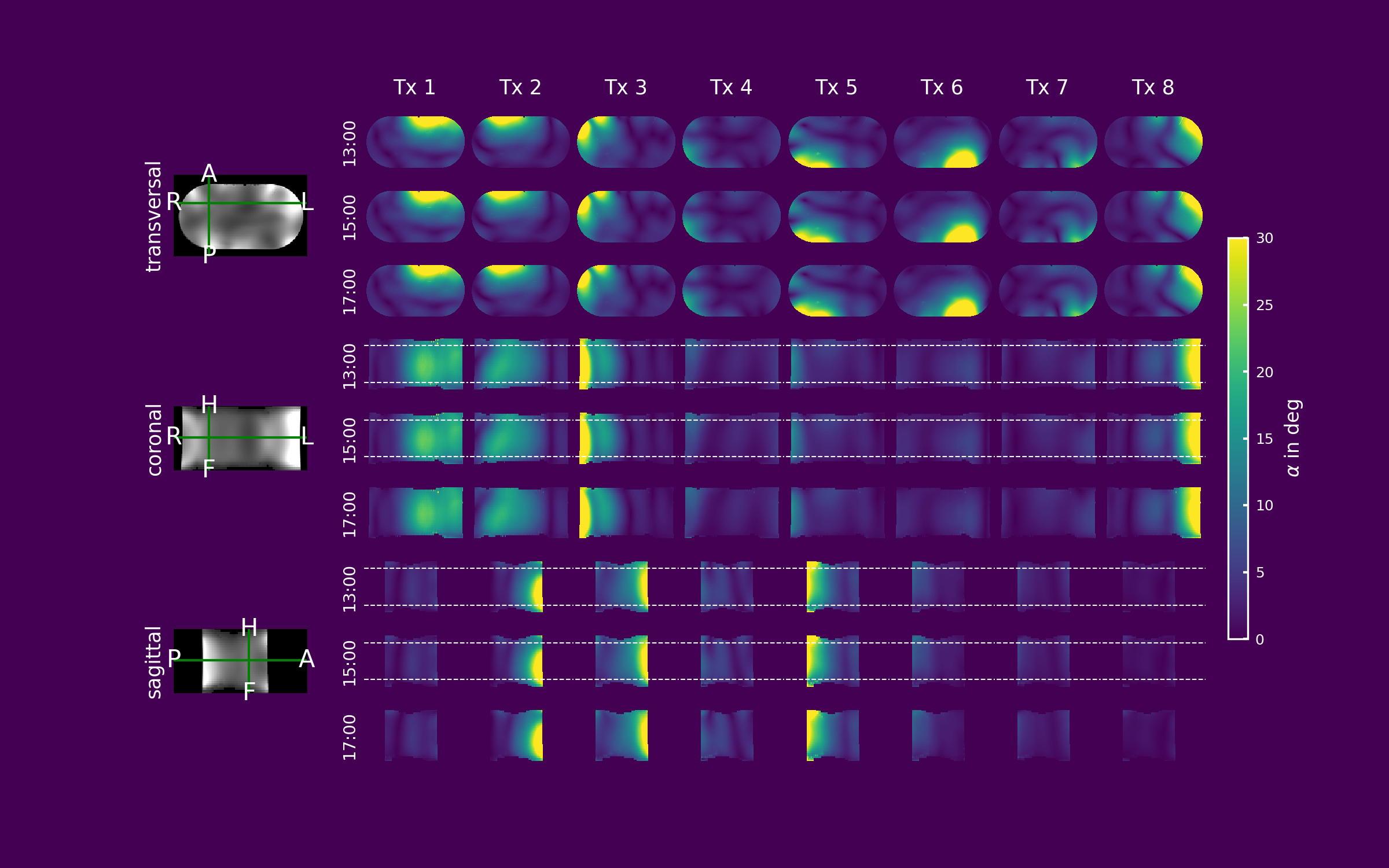


Supporting Information Figure S2: Qualitative results of phantom reproducibility measurements using the same phantom as in Figure 6. Three consecutive scans were performed, with a 2-hour interval between the start of each acquisition. Before each scan, the center frequency was re-adjusted; no further calibration was applied.


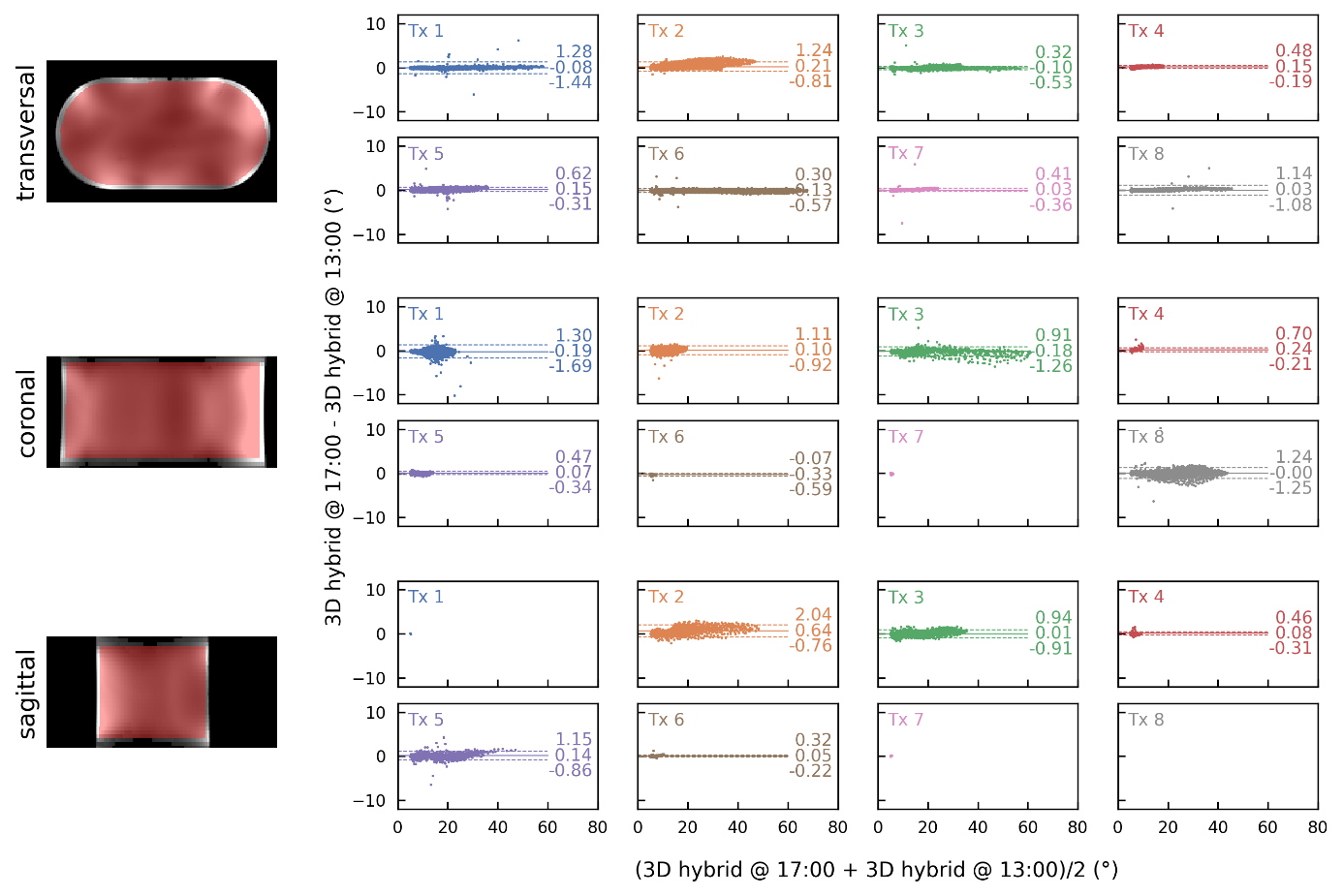


Supporting Information Figure S3: Quantitative evaluation of the reproducibility measurements from Supporting Information Fig.S2, comparing the first and last scan. All data points within the mask (red shaded areas on the left) and with FAs > 5° in both measurements were evaluated. For channels with more than 250 evaluated data points, the mean value is shown and visualized by a solid line, while the ±1.96 SDs from the mean value are shown with dotted lines. Across all evaluated data points, the overall mean±SD of the difference was -0.02±0.49°. For the different orientations and all data points across the different Tx-channels, the mean±SD of the differences were: 0.02±0.44° for the transversal view, -0.05±0.60° for the coronal view, and 0.19±0.56° for the sagittal view.


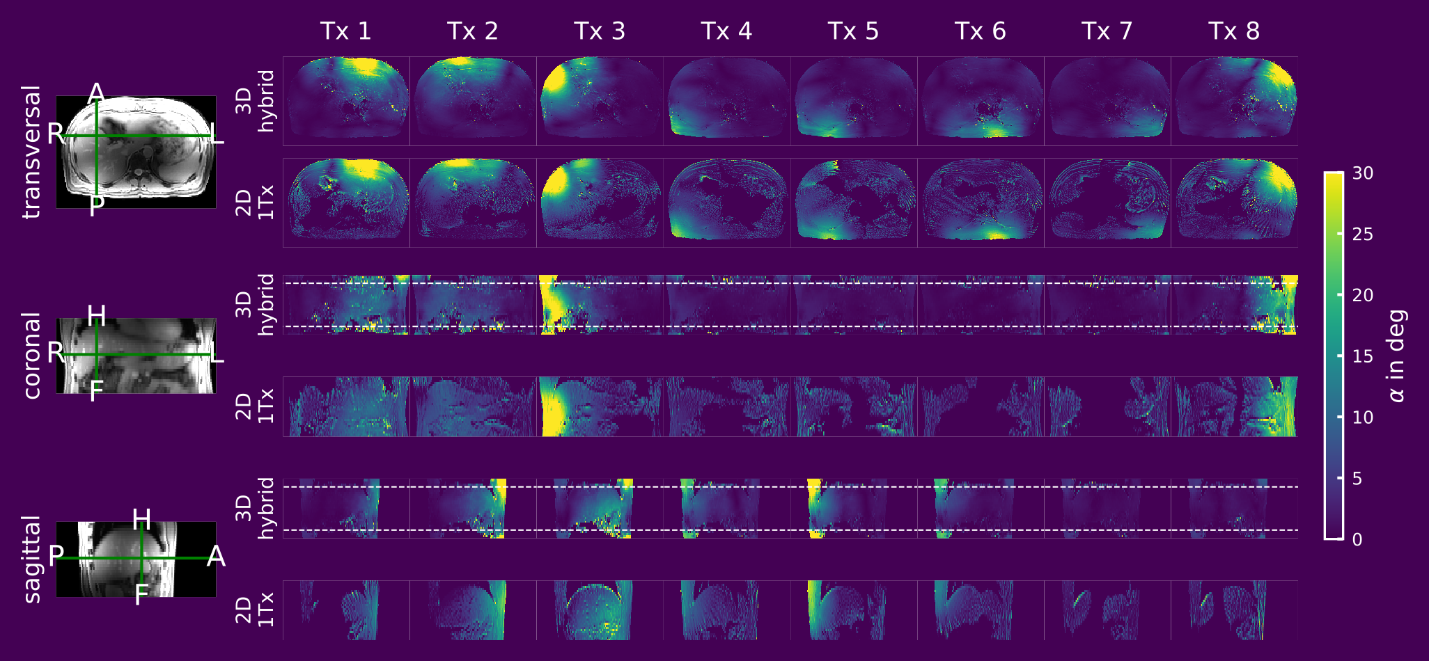


Supporting Information Figure S4: 3D channel-wise hybrid absolute B_1_^+^ maps for an in-vivo scan of a subject with a BMI of 27.8 kg/m². A transversal, coronal, and sagittal slice of the 3D hybrid dataset are shown, alongside the same slices acquired with the 2D B1-MRF sequence using only one Tx at a time. The white dotted line in the 3D measurements indicates the nominal slab thickness.


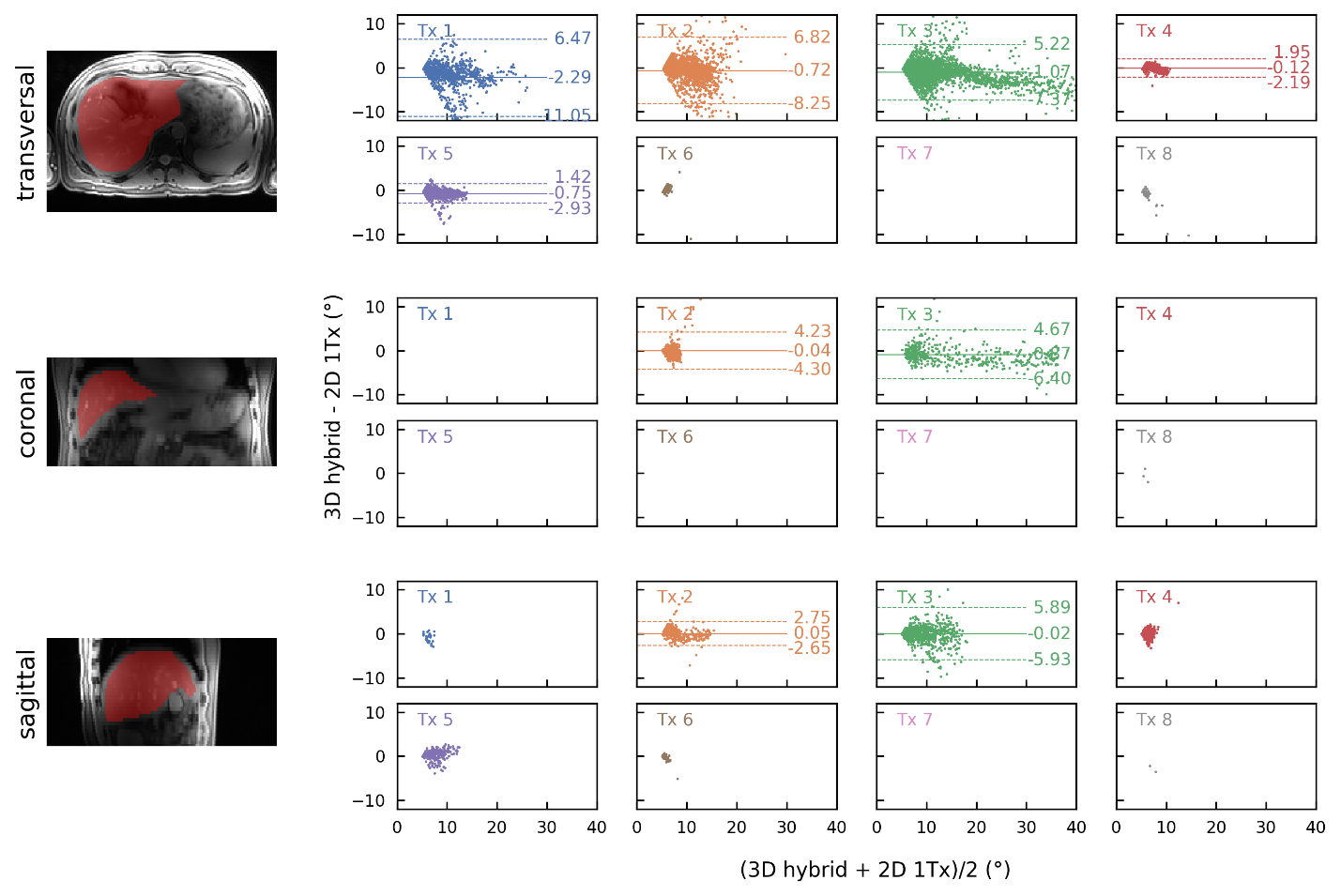
Supporting Information Figure S5: Quantitative comparison of the 3D hybrid approach to the 2D 1Tx measurement in a Bland-Altman plot for the subject with a BMI of 27.8 kg/m² from Supporting Information Figure S4. All data points within the mask (red shaded areas on the left) and with FAs > 5° in both measurements were evaluated. For channels with more than 250 evaluated data points, the mean value is shown and visualized by a solid line, while the ±1.96 SDs from the mean value are shown with dotted lines. Across all evaluated data points, the overall mean±SD of the difference was -0.83±3.20°. For the different orientations and all data points across the different Tx-channels, the mean±SD of the differences were: -1.05±3.40° for the transversal view, -0.47±2.57° for the coronal view, and 0.02±2.27° for the sagittal view.


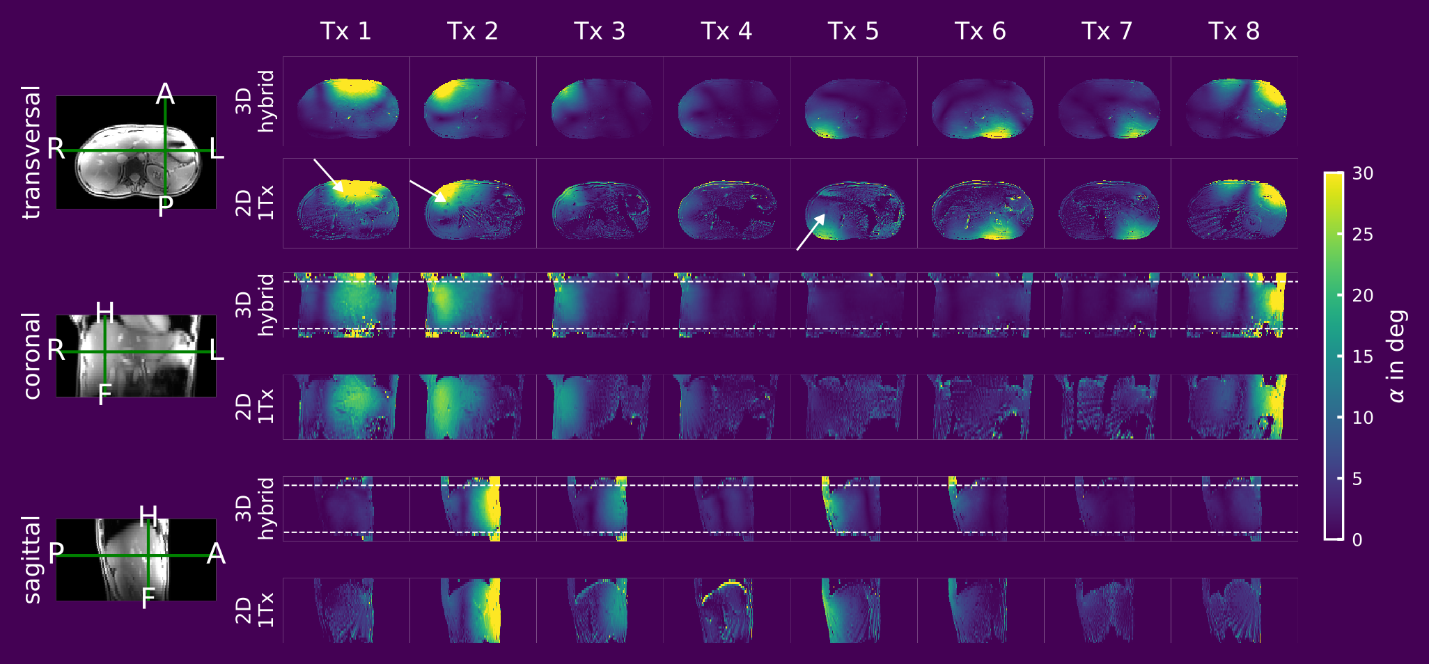


Supporting Information Figure S6: 3D channel-wise hybrid absolute B_1_^+^ maps for an in-vivo scan of a subject with a BMI of 18.1 kg/m². A transversal, coronal, and sagittal slice of the 3D hybrid dataset are shown, alongside the same slices acquired with the 2D B1-MRF sequence using only one Tx at a time. The white dotted line in the 3D measurements indicates the nominal slab thickness. In the transversal 2D 1Tx measurement, flow effects are visible in the maps of the single Tx-channels, which are not present in the 3D hybrid implementation.


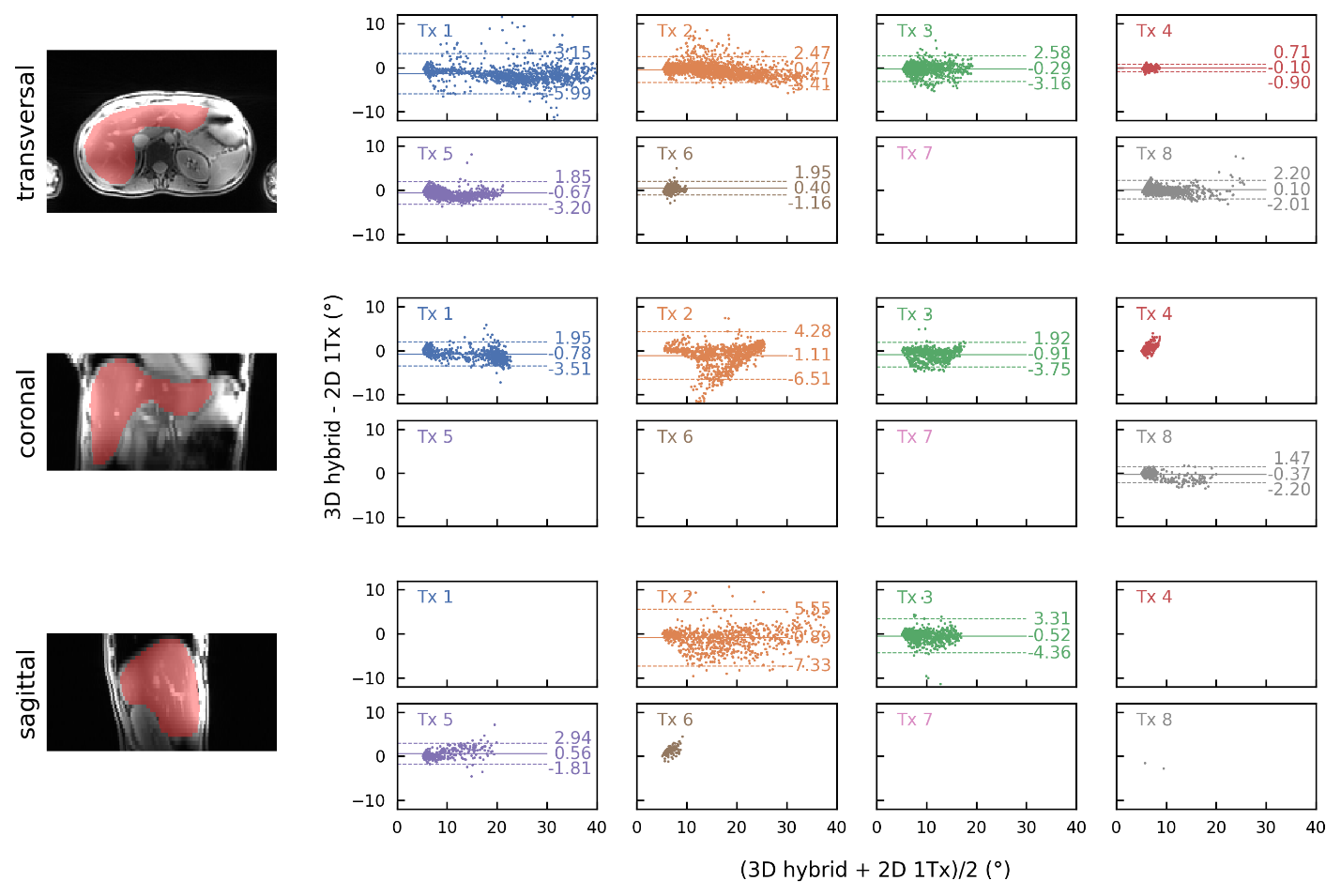
Supporting Information Figure S7: Quantitative comparison of the 3D hybrid approach to the 2D 1Tx measurement in a Bland-Altman plot for the subject with a BMI of 18.1 kg/m² from Supporting Information Figure S6. All data points within the mask (red shaded areas on the left) and with FAs > 5° in both measurements were evaluated. For channels with more than 250 evaluated data points, the mean value is shown and visualized by a solid line, while the ±1.96 SDs from the mean value are shown with dotted lines. Across all evaluated data points, the overall mean±SD of the difference was -0.58±1.97°. For the different orientations and all data points across the different Tx-channels, the mean±SD of the differences were: -0.05±1.63° for the transversal view, -0.78±1.97° for the coronal view, and -0.45±2.28° for the sagittal view.


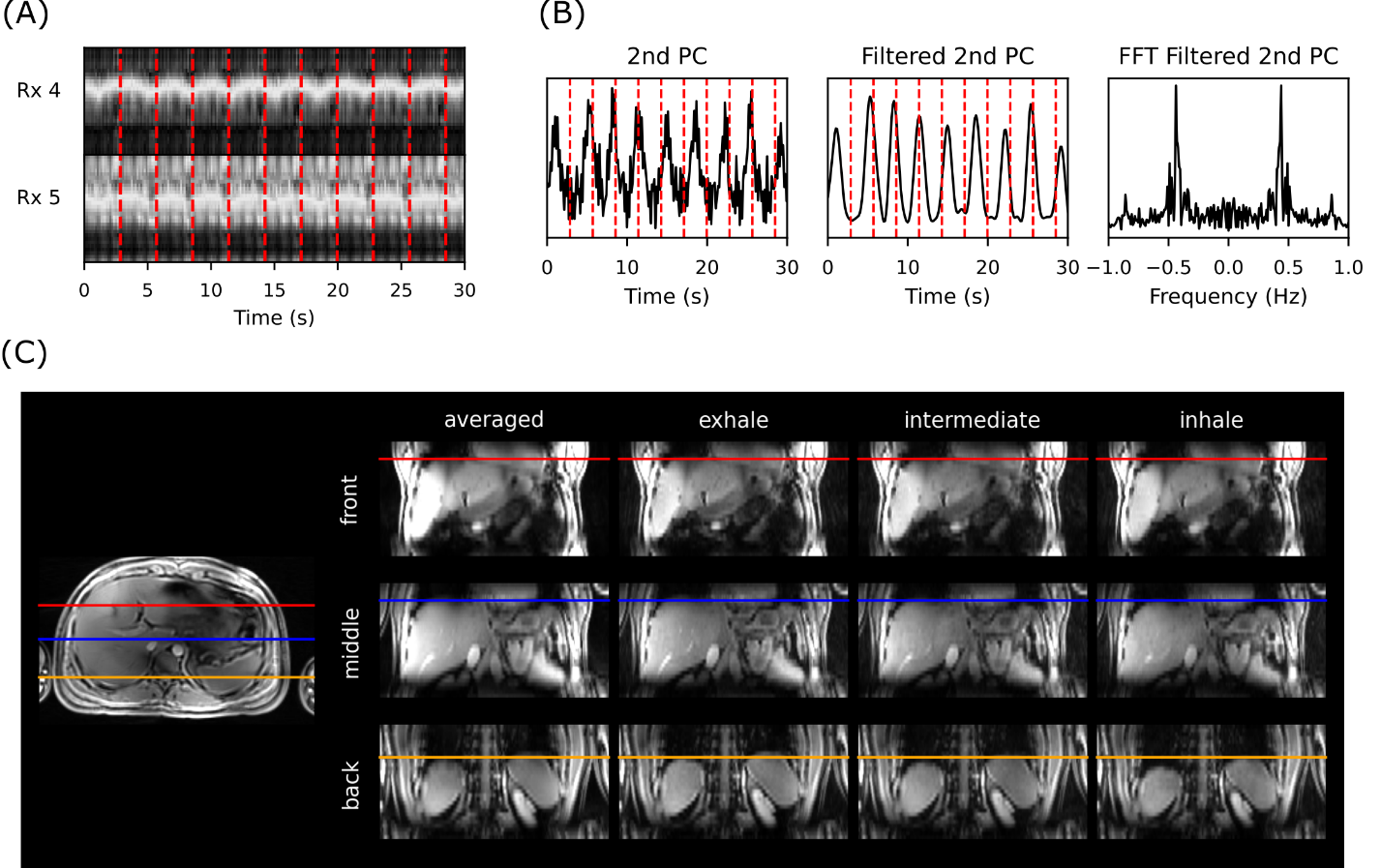


Supporting Information Figure S8: Illustration of the motion-resolved reconstruction. Similar to XD-GRASP^45^, the data is binned into different motion states. (A) A 1D FFT is performed along the k_z_ dimension for k_x_=k_y_=0 for each set. The data is then concatenated into a 2D matrix, where the first dimension represents time and the second dimension represents z+coil. Unlike qualitative MRI, the data at each time point is normalized by its maximum value to suppress contrast changes induced by the applied FA pattern. The red dotted line indicates the start of the next cycle and demonstrates that the signal variations in Rx 4 do not follow the periodicity of the MRF FA pattern. Principal component (PC) analysis was applied to the 2D matrix shown in (A), and the 2nd PC is displayed, as the 1st PC still shows the MRF FA pattern. The signal was low-pass filtered, and the FFT spectrum reveals a peak at 0.44 Hz, which corresponds to the frequency range expected for respiratory motion. (C) displays the reconstruction of data from different motion states for a sagittal orientation at three different positions, marked in the transversal image on the left. Data from different sets and shims are combined, leading to mixed image contrast. In regions of the back, the motion between inhale and exhale shows a detectable effect at the upper border of the liver, while in the middle and front positions, nearly no effect from respiration is observed. Comparing the averaged state to the exhale state at the different coronal positions, the variation along the z-dimension for the top of the liver is less than 1 pixel.


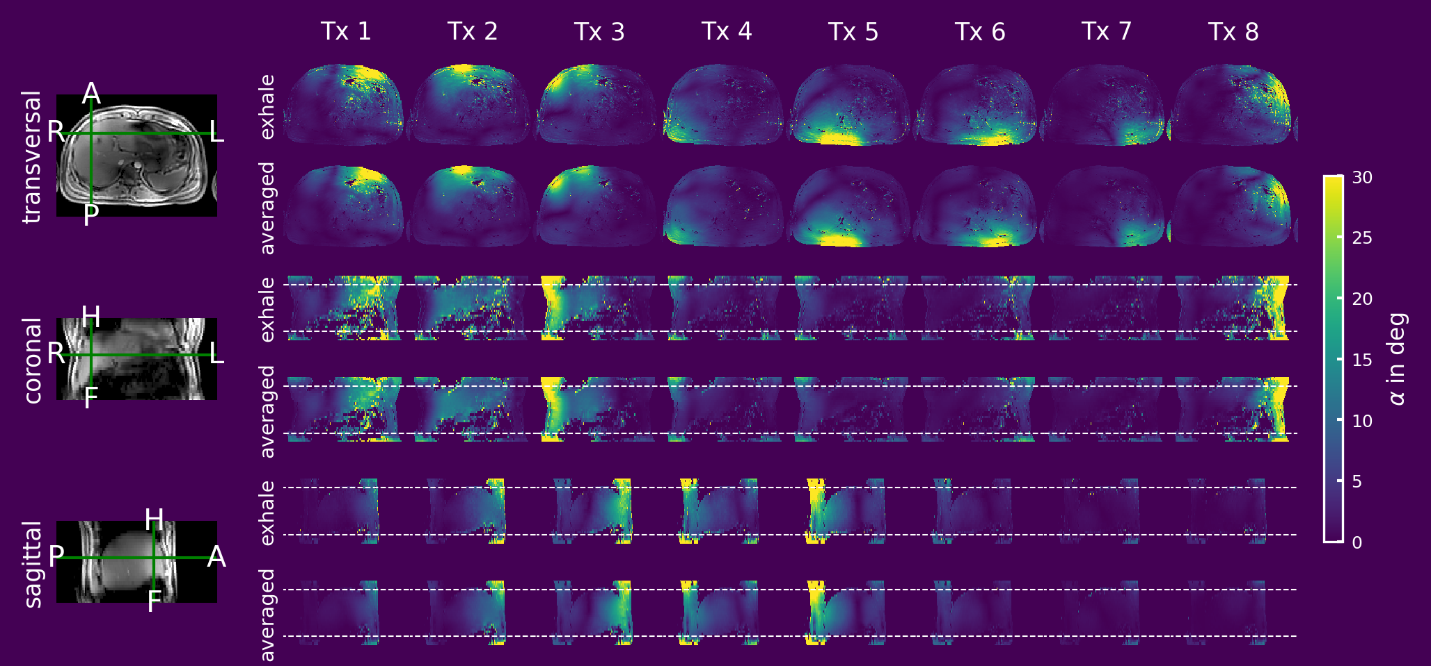


Supporting Information Figure S9: Comparison of the exhale and averaged motion states for the 3D hybrid B1-MRF approach. The acquired data was doubled compared to the parameters stated in Table 2, resulting in double the acquisition time. For the exhaled motion state, which accounted for 40% of the acquired data, the data was binned as shown in Supporting Information Figure S8, and only the data collected during exhalation was used for image reconstruction and MRF matching. In contrast, for the averaged motion state, all data was reconstructed as in the other data presented in this work. In this qualitative comparison, no substantial differences are observed between the averaged and exhaled motion states.


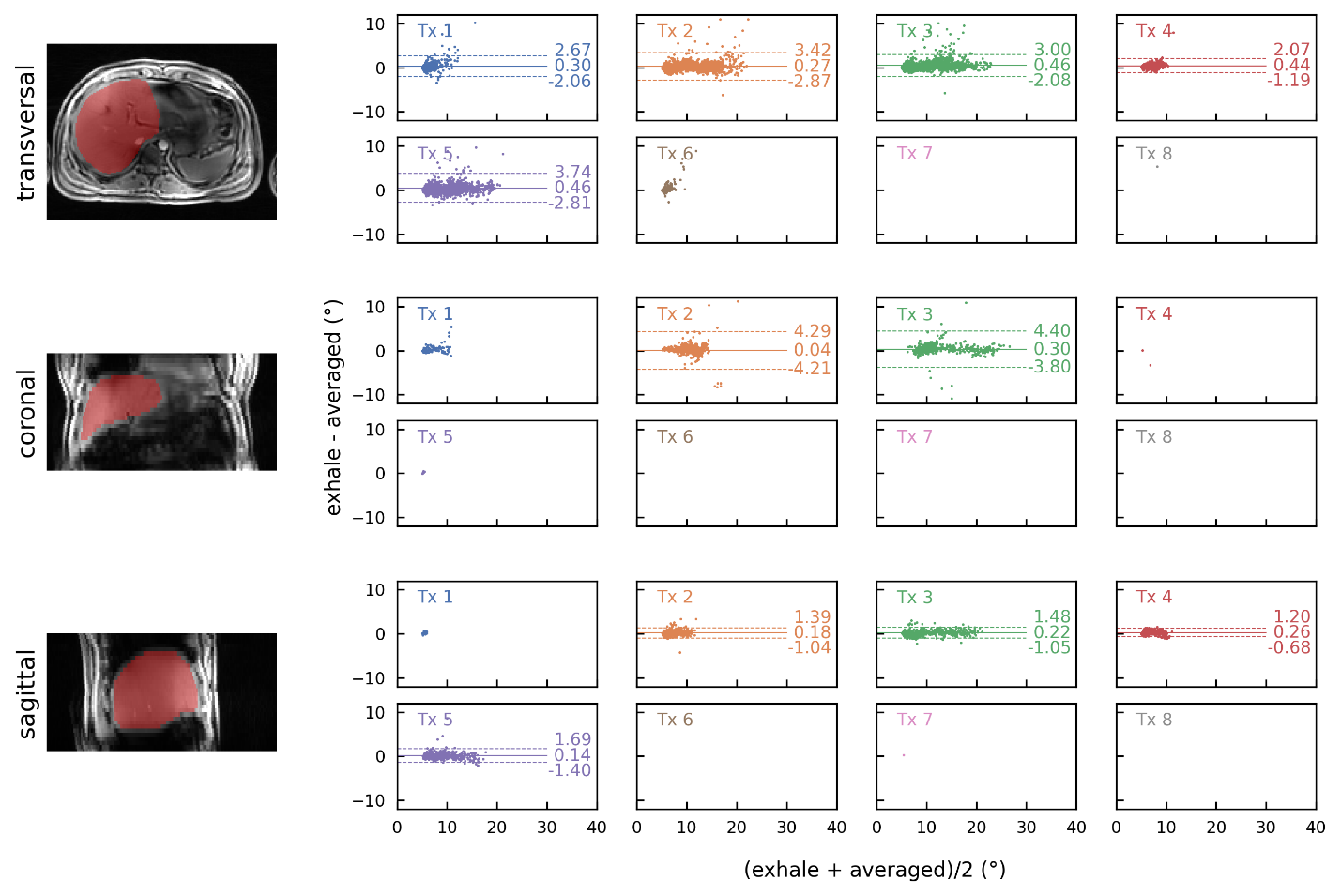


Supporting Information Figure S10: Quantitative comparison of the exhale and averaged motion state in a Bland-Altman plot for the data shown in Supporting Information Figure S9. All data points within the mask (red shaded areas on the left) and with FAs > 5° in both measurements were evaluated. For channels with more than 250 evaluated data points, the mean value is shown and visualized by a solid line, while the ±1.96 SDs from the mean value are shown with dotted lines. Across all evaluated data points, the overall mean±SD of the difference was 0.33±1.45°. For the different orientations and all data points across the different Tx-channels, the mean±SD of the differences were: 0.40±1.44° for the transversal view, 0.18±2.07° for the coronal view, and 0.19±0.65° for the sagittal view.


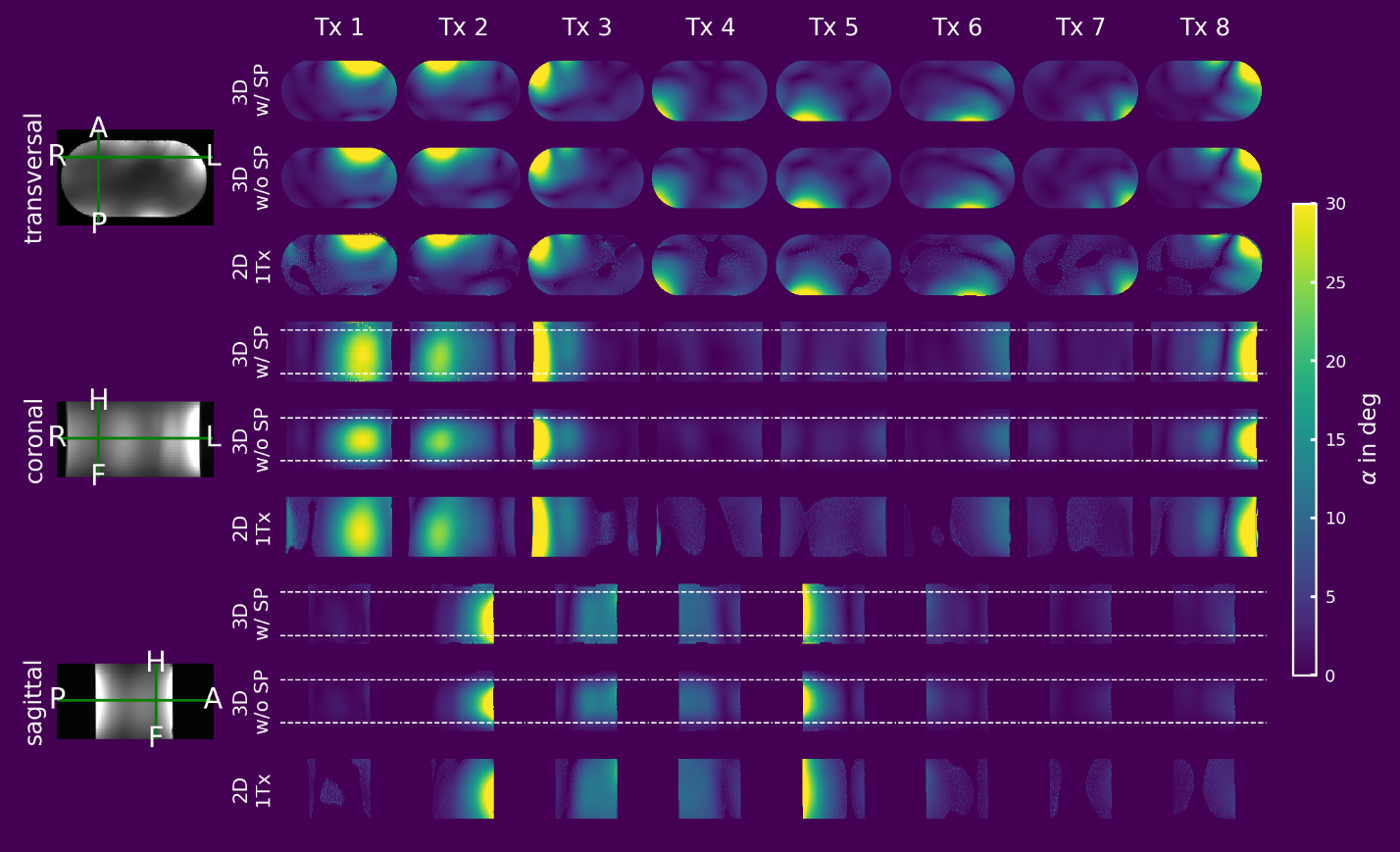


Supporting Information Figure S11: Comparison of including the slab profile in the signal model (w/ SP) versus not including it (w/o SP) for the phantom experiment from Figure 6. No observable difference in the B_1_^+^ results is found for the central slices between w/ SP and w/o SP, and both agree well with the 2D reference. However, in the coronal and sagittal views, not including the slab profile leads to differences that increase with the distance from the slab center. Compared to the 2D reference, substantial differences are observed when the slab profile is excluded in the 3D casefrom the signal model, which are not present when the slab profile is included. Quantitative evaluation within the ROIs drawn in Figure 7 shows that for the transversal orientation at the isocenter, the mean±SD difference to the 2D 1Tx reference remains constant (w/ SP and w/o SP: 0.03±0.67°). However, for the coronal and sagittal orientations, the absolute mean difference increases by a factor greater than 5.0, and the standard deviation increases by a factor greater than 2.6 for the w/o SP reconstruction compared to the w/ SP reconstruction. Specifically, for the coronal view, the mean±SD is w/ SP: 0.59±1.83° and w/o SP: -2.97±4.85°, while for the sagittal view, it is w/ SP: 0.41±1.00° and w/o SP: -2.07±3.55°.


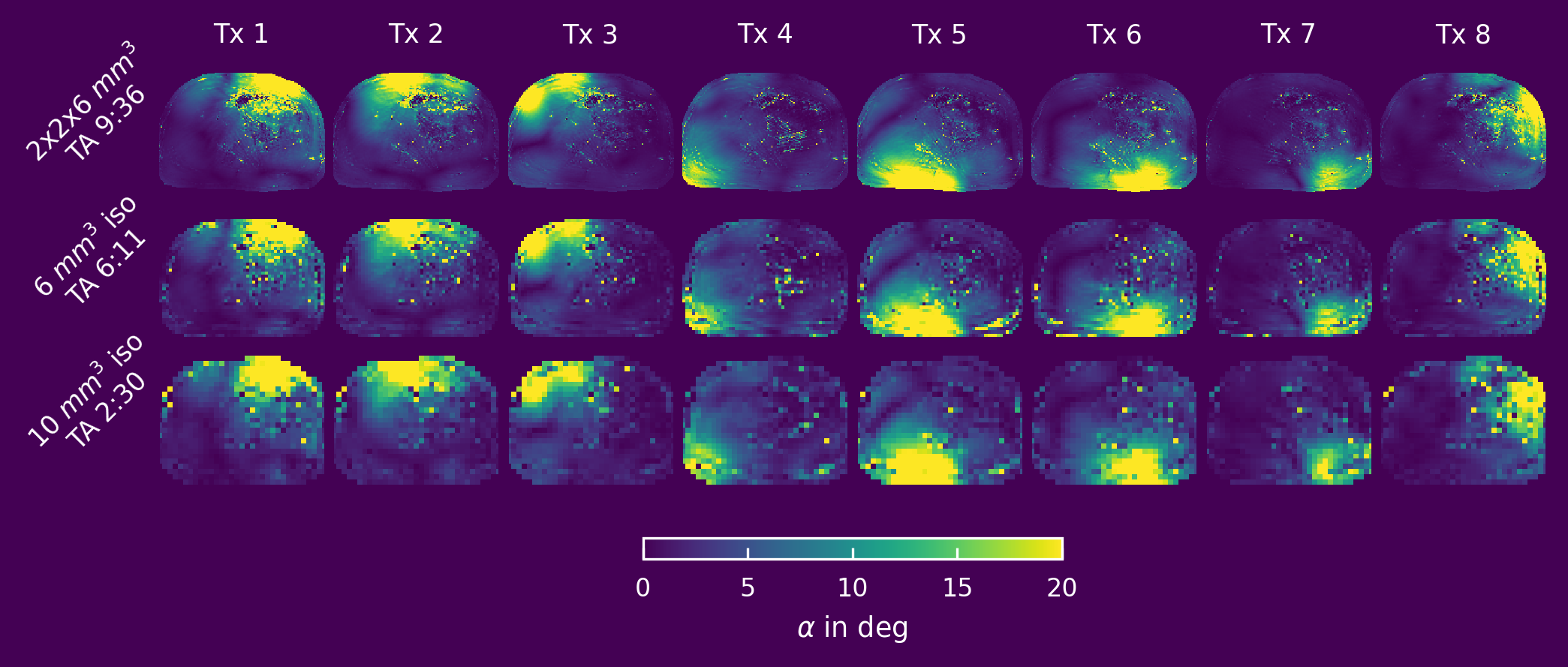


Supporting Information Figure S12: Flexibility of the hybrid 3D B1-MRF method in-vivo. The first row shows the transversal slice at the isocenter with a 2x2x6 mm³ resolution, acquired with a TA of 9 min 36 s, which was used throughout this work. The second row shows a 6 mm³ isotropic resolution, reducing the acquisition time to 6 min 11 s. The last row shows a 10 mm³ isotropic resolution, acquired in 2 min 30 s.
